# Cryo-EM structure of the EBV ribonucleotide reductase BORF2 and mechanism of APOBEC3B inhibition

**DOI:** 10.1126/sciadv.abm2827

**Published:** 2022-04-27

**Authors:** Nadine M. Shaban, Rui Yan, Ke Shi, Sofia N. Moraes, Adam Z. Cheng, Michael A. Carpenter, Jason S. McLellan, Zhiheng Yu, Reuben S. Harris

**Affiliations:** 1 Department of Biochemistry, Molecular Biology, and Biophysics, Institute for Molecular Virology, Masonic Cancer Center, University of Minnesota, Minneapolis, MN 55455, USA.; 2 Janelia Research Campus, Howard Hughes Medical Institute, Ashburn, VA 55416, USA.; 3 Howard Hughes Medical Institute, University of Minnesota, Minneapolis, MN 55416, USA.; 4 Department of Molecular Biosciences, The University of Texas at Austin, Austin, TX 78712, USA.

## Abstract

Viruses use a plethora of mechanisms to evade immune responses. A recent example is neutralization of the nuclear DNA cytosine deaminase APOBEC3B by the Epstein-Barr virus (EBV) ribonucleotide reductase subunit BORF2. Cryo-EM studies of APOBEC3B-BORF2 complexes reveal a large >1000-Å^2^ binding surface composed of multiple structural elements from each protein, which effectively blocks the APOBEC3B active site from accessing single-stranded DNA substrates. Evolutionary optimization is suggested by unique insertions in BORF2 absent from other ribonucleotide reductases and preferential binding to APOBEC3B relative to the highly related APOBEC3A and APOBEC3G enzymes. A molecular understanding of this pathogen-host interaction has potential to inform the development of drugs that block the interaction and liberate the natural antiviral activity of APOBEC3B. In addition, given a role for APOBEC3B in cancer mutagenesis, it may also be possible for information from the interaction to be used to develop DNA deaminase inhibitors.

## Teaser

These studies show how a conserved viral DNA metabolism protein is repurposed to inhibit a potent antiviral factor.

## INTRODUCTION

Ribonucleotide reductases (RNRs) are indispensable for DNA-based organisms by catalyzing the conversion of ribonucleotides to deoxyribonucleotides [reviewed in (*1*, *2*)]. This fundamental activity makes RNRs targets for anticancer, antibacterial, and antiviral therapies [reviewed in (*1*, *3*)]. Viruses augment deoxyribonucleotide supplies by altering pathways that regulate RNR levels in the host, infecting replicating cells that would have high levels of RNR activity or, in the case of many large DNA viruses and bacteriophages, encoding their own RNR [reviewed in (*4*, *5*)]. Herpesvirus RNRs are similar to those in humans, yeast, and some bacteria and belong to the class Ia subgroup consisting of a large and small subunit. The latter RNRs are regulated allosterically by adenosine triphosphate (ATP)/2′-deoxy-ATP (dATP) and by intricate oligomerization mechanisms that ensure a balanced supply of each nucleotide precursor (*1*, *6*, *7*). However, it has been unclear whether viral RNRs use the same regulatory mechanisms, with some evidence suggesting differences and alternative functionalities. For example, viral RNRs are unresponsive to dATP and other effectors that regulate activity (*8*, *9*), β-herpesvirus RNRs lack a gene that encodes a small subunit and thus are presumed inactive (*4*, *5*), and herpes simplex virus 1/2 (HSV-1/2) RNRs have N-terminal extensions that interact with cellular factors unrelated to RNR catalytic activity (*4*, *5*, *10*). Moreover, we recently discovered that the large subunit of the Epstein-Barr virus (EBV) RNR, BORF2, interacts with at least two cellular APOBEC3 enzymes, revealing an unexpected role for herpesvirus RNRs in blocking antiviral innate immunity (*11*, *12*).

The seven-membered APOBEC3 family (A3A to A3D and A3F to A3H) of single-stranded DNA cytosine deaminases elicits broad antiviral activity, often resulting in lethal mutagenesis, with the archetypical example being HIV-1 hypermutation (counteracted by the HIV-1 Vif protein that targets restrictive A3s for polyubiquitination and degradation) [reviewed in (*13*–*15*)]. In recent years, A3 enzymes have also been associated with cancer, as dysregulation of A3B and A3A is linked causally to heavy mutational burdens found in bladder, breast, cervical, head/neck, lung, and other tumor types [reviewed in (*16*–*18*)]. EBV BORF2 and related RNRs from KSHV and HSV-1 are capable of relocalizing these two deaminases from the nuclear compartment into cytoplasmic aggregates, most likely to protect viral genomes from deamination during the lytic phase of DNA replication (*11*, *12*, *19*). In line with this, BORF2 potently inhibits the DNA deaminase activity of A3B (*11*). To date, no eukaryotic viral RNR structures have been determined and the structural basis for RNR interaction with A3 enzymes is unknown. The primary aim of the present study is to determine the structural basis and mechanism for this unanticipated host-pathogen interaction.

## RESULTS

### Cryo–electron microscopy structure of an EBV BORF2-A3Bctd complex

**Fig. 1.**
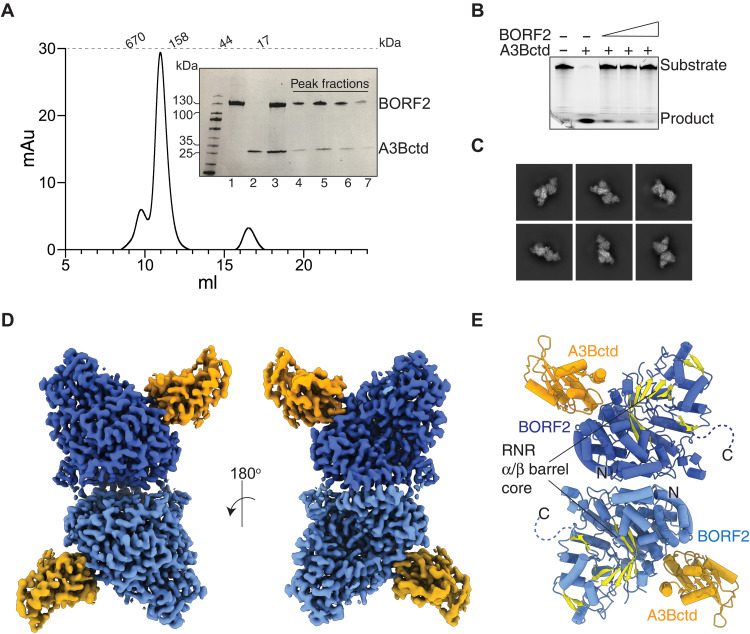
Structure of EBV BORF2-A3Bctd complex. (**A**) Size exclusion chromatogram for BORF2-A3Bctd complex. Inset: SDS–polyacrylamide gel electrophoresis gel of MBP-BORF2, A3Bctd-mycHis, the complex before SEC (lanes 1 to 3), and fractions of the major peak (lanes 4 to 8). (**B**) BORF2 inhibits A3Bctd single-stranded DNA deaminase activity. Negative control is substrate alone, positive control is A3Bctd alone, and dose-responsive inhibition occurs with increasing BORF2 concentrations. (**C**) Representative 2D classes of the BORF2-A3Bctd complex. (**D**) Cryo-EM composite map of BORF2-A3Bctd complex, with BORF2 in blue and A3Bctd in orange. (**E**) Ribbon schematic of the BORF2-A3Bctd complex, with the RNR core depicted in yellow.

Previous studies indicated that the BORF2-A3B interaction requires the BORF2 RNR core domain and the A3B C-terminal catalytic domain (ctd) (*11*). We therefore focused on forming a complex between BORF2 and A3Bctd. Initial attempts to purify BORF2 from *Escherichia coli* were unsuccessful. However, expression of BORF2 in 293T cells with an N-terminal maltose binding protein (MBP) tag substantially improved solubility, purity, and yield (Fig. 1A). Catalytically active A3Bctd with a C-terminal myc-His tag was also expressed in 293T cells (Fig. 1A). The purified BORF2 protein inhibits A3B catalytic activity in vitro (Fig. 1B), consistent with a previous study (*11*). To form a BORF2-A3B complex, BORF2 and A3Bctd were purified individually with affinity resins, mixed together with excess A3Bctd, and isolated as a complex by size exclusion chromatography (SEC) (Fig. 1A). This complex migrated at a size consistent with a higher-order assembly and a 1:1 BORF2:A3Bctd ratio (Fig. 1A).

Cryo–electron microscopy (cryo-EM) was used to determine the structure of the BORF2-A3Bctd complex. A total of 7488 multiframe movies were collected using a Titan Krios with a post–energy filter K3 detector and processed in cryoSPARC (*20*). Two-dimensional (2D) classes corresponding to what appeared to be dimeric forms of the complex were selected for 3D reconstruction and heterogeneous refinement yielding a 2.8-Å map. 3D variability analysis was performed to assess the conformational flexibility of the complex (*21*). Movement was particularly detected at the BORF2 dimer interface (movies S1 and S2). Therefore, a focused refinement around the BORF2-A3B monomer was performed after symmetry expansion that resulted in an improved reconstruction to 2.55 Å. A composite map to represent the dimeric form of the complex was generated from the focused refined map (figs. S1 and S2 and table S1). The cryo-EM maps allowed the modeling of nearly all components of the BORF2-A3Bctd complex with higher-resolution features at the BORF2 catalytic core and the BORF2-A3B interface (fig. S2). The structure is composed of a dimer of BORF2 monomers, which are each bound to one A3Bctd protomer (Fig. 1, C to E).

### Conserved features of EBV BORF2 with respect to other class Ia RNRs

**Fig. 2.**
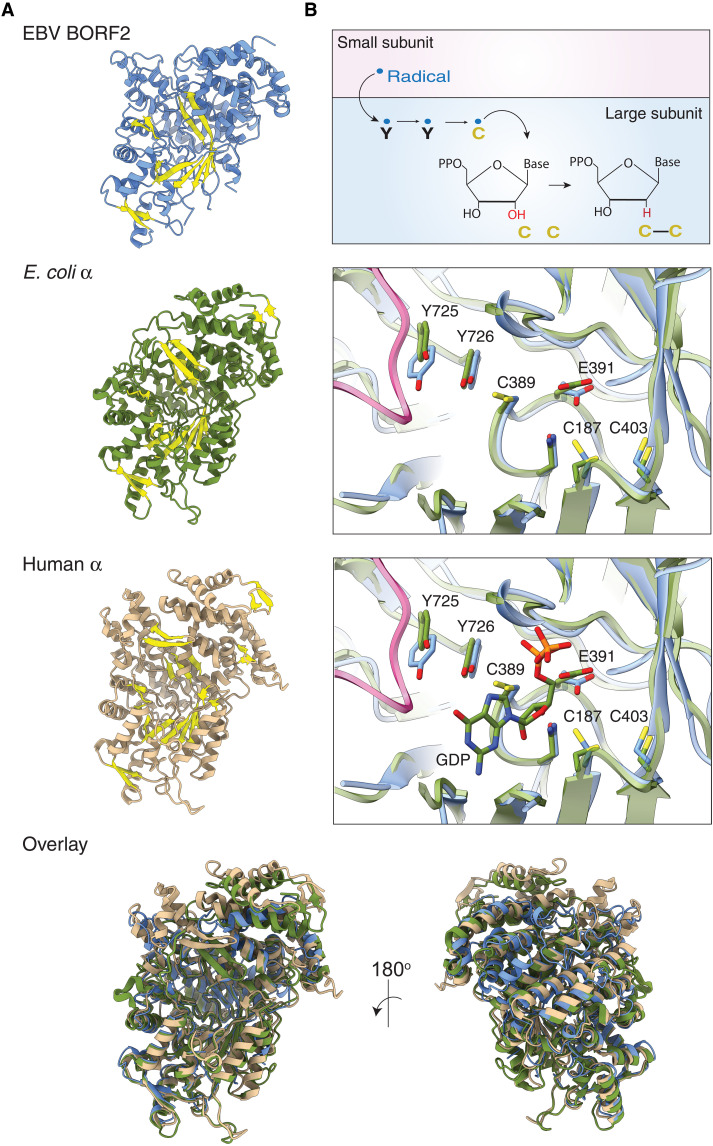
RNR large subunit structural conservation. (**A**) EBV BORF2 (blue), *E. coli* RNR α subunit [Protein Data Bank (PDB): 6w4x; green], and human RNR α subunit (PDB: 6aui; tan) share a conserved α/β barrel catalytic core and overall architecture. β strands are highlighted in yellow for visual comparison, and an overlay is shown below. (**B**) Cartoon schematic of the proposed radical-based mechanism for ribonucleotide reduction with coordination between the small (pink) and large (blue) subunits. Zoom-in of the overlay of the active-site regions of EBV BORF2 (blue) and *E. coli* RNR α subunit (PDB: 6w4x, chain B; green), showing conservation of the tyrosine and cysteine residues involved in radical transfer and oxidation. Residue numbers correspond to BORF2.

Although sharing only ~25% primary amino acid identity, the structure of BORF2 overlays well with the RNRs of *E. coli* and humans with root mean square deviation of 1.1 and 1.2 Å, respectively (Fig. 2A). BORF2 adopts an α/β barrel RNR architecture resembling the large subunit of all classes of RNRs (Figs. 1E and 2A) (*22*). Particularly high conservation is observed for residues involved in ribonucleotide reduction (Fig. 2B). For class I RNRs, ribonucleotide reduction involves the transfer of a radical generated in the iron-coordinating small subunit of the RNR to the large subunit via tyrosine and cysteine amino acid radical intermediates (*1*). The resulting thiyl radical formed in the large subunit initiates the reduction of the 2′-OH of the ribonucleotide and subsequent oxidation of a pair of redox-active cysteines positioned on adjacent strands. BORF2 residues Y725, Y726, C389, C187, and C403 overlay with the established catalytic residues of other class Ia RNRs involved in this process (Fig. 2B). The predicted redox-active cysteines (C187 and C483) are in a reduced state in the BORF2 structure. The C-terminal end of BORF2 predicted to contain additional cysteines required for postcatalytic re-reduction of the active-site cysteines is disordered similar to other class Ia RNRs [e.g., (*23*–*25*); dashed line to C-terminus in Fig. 1E].

### Unique features of the EBV large RNR subunit BORF2

**Fig. 3.**
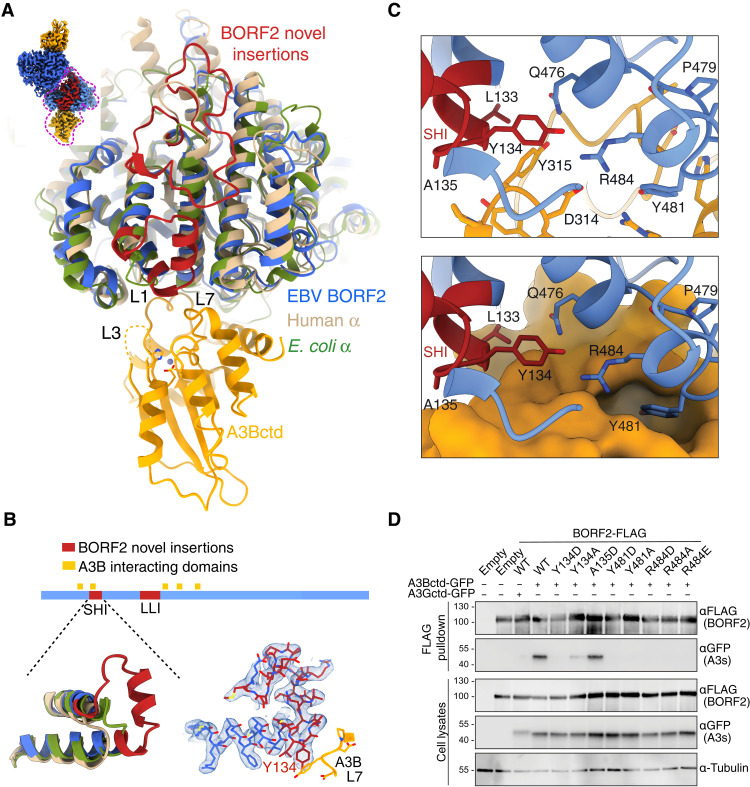
Unique features of EBV BORF2 and its interaction with A3B. (**A**) Overlay of the structures of BORF2 (blue), human RNR α subunit (PDB: 6aui, chain A; tan), and *E. coli* RNR α subunit (PDB: 6w4x, chain B; green). Novel BORF2 insertions SHI and LLI are depicted in red. Loop regions surrounding the A3Bctd active site are labeled L1, L3, and L7. (**B**) Schematic of BORF2 (1 to 826 amino acids) showing the A3B interacting regions (orange) and the novel SHI and LLI insertions (red). Bottom left: Structural comparison of the BORF2 SHI and the corresponding regions of human and *E. coli* RNR α subunits. Bottom right: Cryo-EM map of the BORF2 SHI domain in blue. (**C**) Close-up of BORF2-A3B interface showing a network of interactions. BORF2 is depicted in blue, BORF2 SHI is in red, and A3Bctd is in orange, with ribbon and surface representations (top and bottom, respectively). (**D**) Co-IP reactions with the indicated BORF2 constructs (anti-FLAG) and A3Bctd-eGFP. A parallel reaction with BORF2 and A3Gctd-eGFP is shown as an additional negative control. Input blots are shown below including anti-tubulin as a loading control.

The presence of two novel insertions in EBV BORF2 distinguishes this viral enzyme from all other class Ia RNRs (red in Fig. 3, A to C, and fig. S3). First, a short-helix insertion (SHI) is part of an extended loop that packs between four larger structurally conserved helices in BORF2. This novel insertion contributes multiple residues in binding A3B (Fig. 3, A to C, and below). For instance, BORF2 SHI residues L133 and Y134 directly contact A3Bctd (Fig. 3, B and C, and fig. S3, A to C). Y134 additionally forms a network of stabilizing interactions with other BORF2 residues including Q476 and R484. The importance of Y134 is evidenced by coimmunoprecipitation (co-IP) experiments in which a Y134D substitution abrogates the interaction and a Y134A substitution weakens it (Fig. 3D). Second, a long-loop insertion (LLI) anchored on the RNR core has interactions with the SHI and surrounding helices (red in Fig. 3A and fig. S3, A to C). In particular, LLI directly contacts the SHI, which, in turn, binds to A3B (fig. S3, B and C). It is therefore possible that the LLI is also essential for the interaction with A3B, and this and other potential longer-range interactions will be a focus of future studies.

Next, we asked whether related herpesvirus RNRs have homologous insertions or whether the SHI and LLI are unique to EBV BORF2. The KSHV RNR large subunit ORF61 and the HSV-1 RNR large subunit ICP6 (UL39) have also been shown to bind A3s (*11*, *12*, *19*). However, amino acid sequence alignments of BORF2, ORF61, and ICP6 indicated limited identity in the SHI region and deletions in the LLI region (fig. S4). We therefore used AlphaFold (*26*) to generate structural models for 3D comparisons. The AlphaFold model of BORF2 has an overall organization and structure that is similar to our cryo-EM structure (fig. S4). This constitutes independent validation of our cryo-EM studies and indicates that comparative modeling using AlphaFold may be informative for viral RNRs. It is therefore interesting that AlphaFold predicts that both KSHV ORF61 and HSV-1 ICP6 have a similar-sized short insertion but lack an analogous large insertion (fig. S4). Together, the SHI appears unique to EBV BORF2 and potentially also to large RNR subunits of related herpesviruses known to bind A3 enzymes. We therefore postulate that the SHI may have been an evolutionary adaptation of an ancestral herpesvirus that provided a competitive advantage by neutralizing A3 DNA deaminase activity and helping to create an environment permissive for lytic DNA replication.

### EBV BORF2 binds selectively to loops 1 and 7 of the A3B catalytic domain

**Fig. 4.**
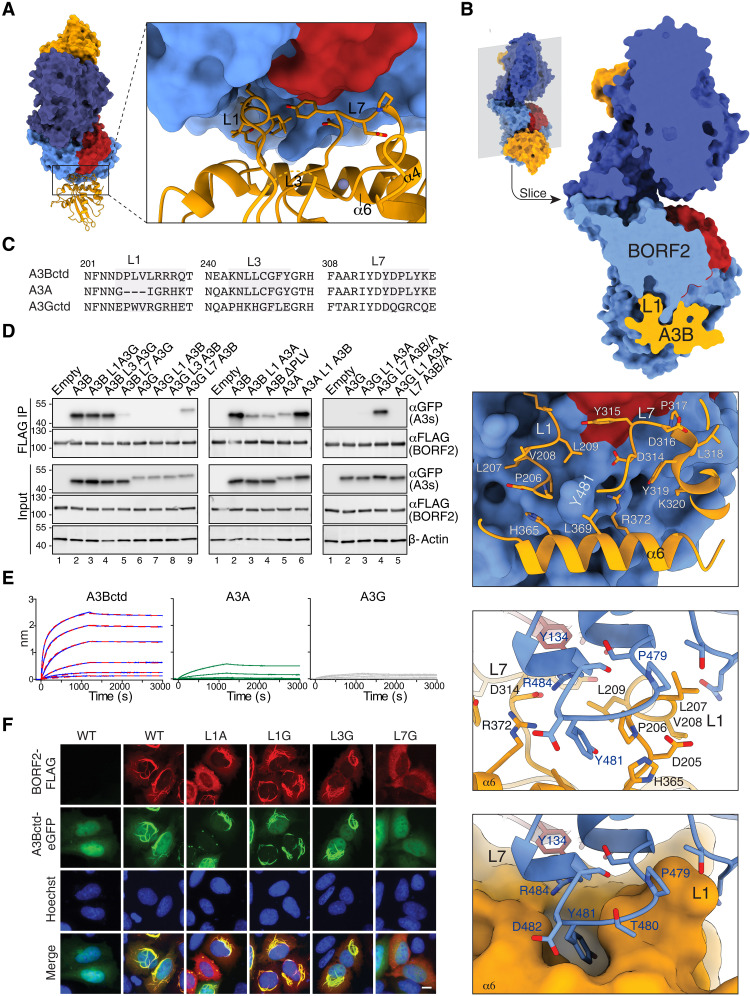
EBV BORF2 binds selectively to A3Bctd loops 1 and 7. (**A**) A surface representation of the BORF2-A3Bctd complex with one of the A3Bctd protomers represented in ribbons. Right: Close-up of the interacting surfaces highlighting BORF2 SHI (red) and A3Bctd L1, L7, and α6 regions (orange). (**B**) Cross-sectional representation of the BORF2-A3Bctd complex showing deep interlocking contacts. The enlargements below, for instance, show BORF2 Y481 inserted between the L1 and α6 regions of A3Bctd. The bottom images are stick and surface filled representatives showing an alternative angle, respectively. (**C**) Amino acid alignment of the L1, L3, and L7 regions of A3Bctd, A3A, and A3Gctd (full alignments in fig. S5). (**D**) BORF2 (anti-FLAG) co-IP experiments with A3Bctd-eGFP, A3A-eGFP, and A3Gctd-eGFP proteins in comparison to the indicated loop swap variants. Input blots are shown below including anti–β-actin as a loading control. (**E**) BLI sensorgrams of BORF2 binding to A3Bctd-mycHis, A3A-mycHis, and A3G-mycHis immobilized on Ni–nitrilotriacetic acid probes. The BORF2-A3Bctd interaction has a calculated dissociation constant (*K*_d_) of 0.78 based on fitting data with a 1:2 binding model (red dashed line represents fit of the curves to model). (**F**) Representative fluorescence microscopy images of BORF2-FLAG and the indicated A3Bctd-eGFP constructs. Nuclei are stained with Hoechst. Scale bar, 10 μm.

All A3s share a common cytidine deaminase fold in which the active site is composed of a zinc-coordinating domain surrounded by three loops—loop 1 (L1), loop 3 (L3), and loop 7 (L7)—that combine to engage substrate single-stranded DNA [(*27*–*29*) and reviewed in (*30*, *31*); fig. S5]. These loops vary in size and amino acid composition and account for differences in enzymatic properties including catalytic rates and local sequence preferences [reviewed in (*30*, *31*)]. Our previous studies implicated A3Bctd L7 in the interaction with BORF2 (*11*). Our cryo-EM studies provide an elegant structural explanation for this interaction, with the majority of L7 making direct contact with BORF2 (Fig. 4, A and B). However, the cryo-EM structure also revealed an unanticipated and equally extensive interaction between EBV BORF2 and the L1 region of A3Bctd (Fig. 4, A and B). BORF2 residue Y481 is inserted in a hydrophobic pocket generated by A3B L1 residues 206-PLV-208 and residues H365 and L369 of the α6 region (Fig. 4B and fig. S6A). The interlocked positioning of BORF2 Y481 is likely aided by a neighboring proline (P479) that kinks the BORF2 loop containing these residues (fig. S6, A to C).

To validate the importance of these loops, a panel of A3B catalytic domain loop swap chimeras was tested in co-IP experiments (A3A, A3Bctd, and A3Gctd loop alignments in Fig. 4C). As shown previously (*11*), BORF2 strongly coimmunoprecipitates A3Bctd but not A3Gctd (Fig. 4D, left, lane 2 versus lane 6). Replacing L7 of A3Bctd with L7 of A3Gctd nearly abolished the interaction (left, lane 2 versus lane 5), and reciprocally, replacing L7 of A3Gctd with L7 of A3Bctd strengthened the interaction (left, lane 6 versus lane 9). Similarly, when L1 of A3A is grafted into A3Bctd, or when amino acids 206-PLV-208 are deleted from A3Bctd L1 to make it more A3A-like, marked decreases in A3Bctd co-IP are observed (Fig. 4D, middle, lanes 2 to 5). Reciprocally, when L1 of A3Bctd is used to replace L1 of A3A, a clear increase in pulldown strength becomes evident (Fig. 4D, middle, lanes 5 and 6). Moreover, the strong interaction between BORF2 and A3Gctd containing L7 of A3Bctd/A3A is abrogated by replacing the L1 region of this chimera with L1 of A3A (Fig. 4D, right, lanes 4 and 5). This can be explained by similarities between A3B and A3G L1 regions where these enzymes both have a three-residue motif that is absent in A3A (PLV and PWV, respectively; Fig. 4, C and D, and fig. S5). These differential co-IP results are supported by binding studies in which the BORF2-A3Bctd interaction has a biolayer interferometry (BLI) dissociation constant of ~1 nM, the BORF2-A3A interaction is substantially weaker, and the BORF2-A3G interaction is nearly undetectable (Fig. 4E). Together, these results demonstrate the critical roles of these two loop regions and also show that BORF2 binds preferentially to A3B.

Specific residues within the BORF2-A3B interface were also interrogated by co-IP experiments with single amino acid substitution mutants (Fig. 3D and fig. S6D). BORF2 residues Y134 and L133 (SHI domain) interact directly with Y315 in L7 of A3Bctd, and BORF2 residue R484 contacts D314 of L7 A3Bctd. To reiterate a point from above, the importance of BORF2 Y134 is supported by substitutions that abolish (Y134D) or weaken the interaction (Y134A). Likewise, the importance of BORF2 R484 is shown by several single amino acid substitution mutations that abrogate the interaction (R484A, R484D, and R484E). On the A3B side of the complex, L7 single amino acid substitutions strongly (Y315D) or moderately (D316Q and P317G) disrupt the interaction (fig. S6D). Similarly, a deletion of the PLV motif in L1 of A3Bctd also abrogates the interaction (fig. S6D).

### Relocalization phenotypes of A3B catalytic domain loop swap chimeras

A notable feature of the BORF2-A3B interaction is that it results in relocalization of normally nuclear A3B into large cytoplasmic aggregates (*11*, *12*). We therefore next compared the relocalization phenotypes of the panel of A3Bctd loop swap chimeras described above. This was done by cotransfecting HeLa cells with relevant constructs and, the next day, using fluorescence microscopy to analyze subcellular localization phenotypes. BORF2 causes wild-type A3Bctd to form into large oblong cytoplasmic aggregates (representative images in Fig. 4F and additional images and statistics in fig. S7A). Replacing A3Bctd L1 with the corresponding loop region from A3Gctd had no effect, as expected by the similarity between these loops (above). L3 from A3Gctd also had no effect. Moreover, as expected from the co-IP results above, replacing the L7 region of A3Bctd with the corresponding loop region from A3Gctd prevented BORF2 from triggering cytoplasmic aggregates. However, in contrast to the co-IP results above, which diminished the BORF2-A3Bctd interaction, replacing A3Bctd L1 with the corresponding L1 region from A3A showed no change in relocalization.

To further investigate these effects, a corresponding series of A3A and A3Gctd loop swap chimeras was analyzed by fluorescence microscopy (representative images and statistics in fig. S7, B and C). In contrast to the co-IP results above, BORF2 promotes an A3Bctd-like cytoplasmic aggregation phenotype for A3A that is not further increased by replacing the L1 region with the corresponding loop region of A3Bctd or A3Gctd. However, like A3Bctd, swapping the L7 region of A3A with the corresponding L7 region of A3Gctd strongly disrupted the cytoplasmic aggregation phenotype. Conversely, BORF2 was only able to mediate the relocalization of an A3Gctd construct with the L7 region from A3A/B. These microscopy results combine to underscore the importance of the L7 region in mediating the interaction between BORF2 and A3B and, together with the co-IP results above, suggest that L1 may have a supporting role in strengthening the interaction (Discussion).

### BORF2 forms a noncanonical dimer

**Fig. 5.**
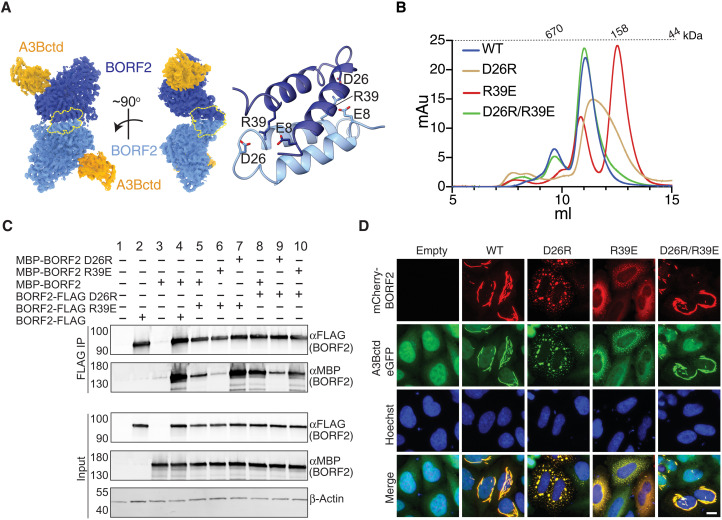
Noncanonical EBV BORF2 dimerization interface influences A3Bctd localization pattern. (**A**) Side and front views of the BORF2-A3Bctd structure with a zoom-in of the four-helix bundle dimer interface. The dimer is mediated, in part, by interactions between R39 and E8 of one monomer and D26 of the opposing monomer. (**B**) Size exclusion profiles of wild-type, D26R, R39E, and D26R/R39E BORF2 proteins. (**C**) Co-IP reactions with the indicated BORF2-FLAG and MBP-BORF2 constructs. Input blots are shown below including anti–β-actin as a loading control. Schematics above depict key interactions between BORF2 monomers. (**D**) Representative fluorescence microscopy images of A3Bctd-eGFP and the indicated mCherry-BORF2 constructs. Nuclei are stained with Hoechst. Scale bar, 10 μm.

The N-terminus of human, *E. coli*, and several class Ia RNRs has an evolutionarily conserved mobile ATP cone domain that regulates activity (ATP activates, whereas dATP inhibits) [reviewed in (*1*, *32*); fig. S8]. The majority of this cone domain is absent in BORF2 including the ATP binding residues, and instead, the N-terminus adopts a helix that mediates formation of a noncanonical and novel dimer (Fig. 5A). This dimer is driven by a leucine-rich hydrophobic interaction involving two α helices from each monomer (i.e., a four-helix bundle) and electrostatic interactions including residues R39, D26, and E8 (Fig. 5A). R39 from each monomer directly interacts with E8 from the same monomer and D26 from the opposing monomer. SEC data support the likelihood that this noncanonical dimerization surface is relevant, as a BORF2 R39E single amino acid mutant eluted slower and at a size consistent with a monomer in comparison to wild-type BORF2 (Fig. 5B). BORF2 D26R showed an intermediate elution profile consistent with partial function. The double mutant R39E/D26R, which is predicted to have restored electrostatics based on the cryo-EM structure, eluted at a SEC position nearly indistinguishable from the wild-type protein.

To further investigate whether BORF2 is capable of dimer formation at this noncanonical interface, we coexpressed BORF2 harboring two different tags (N-terminal MBP and C-terminal 3xFLAG) and performed anti-FLAG co-IP assays, which resulted in a pulldown of a complex containing both tagged forms of BORF2 (Fig. 5C). As predicted by the structure, single amino acid substitutions of R39E or D26R weaken the interaction, and this defect could be reversed fully by combining these changes into a double mutant (R39E/D26R) (e.g., compare lanes 4 to 7 in Fig. 5C).

We next asked whether BORF2 dimerization influences the cytoplasmic aggregation phenotype shown above in Fig. 4F. Pre-engineered HeLa T-REx cells were transfected with mCherry-BORF2, mCherry-BORF2 R39E, mCherry-BORF2 D26R, and mCherry-BORF2 D26R/R39E; treated 24 hours later with doxycycline to induce enhanced green fluorescent protein (eGFP), A3Bctd-eGFP, or full-length A3B-eGFP; and analyzed by immunofluorescence (IF) microscopy after an additional 24 hours of incubation. Wild-type BORF2 relocalizes both A3Bctd and full-length A3B from the nuclear compartment to the cytoplasm where aggregates accumulate (representative images in Fig. 5D and additional images and statistics in fig. S9). BORF2 D26R depletes A3B from the nucleus but forms smaller cytoplasmic structures, consistent with partial function and the SEC results described above. In contrast, R39E retains the ability to relocalize A3B from the nucleus but has a strongly reduced ability to form these structures. However, the D26R/R39E double substitution restores BORF2’s ability to form these filament-like structures, further confirming a direct interaction between these residues. Similar results were obtained for a D26R/R39D double mutant (representative images in fig. S10). These data indicate that noncanonical BORF2 dimerization facilitates the formation of higher-order BORF2-A3B aggregates in cells.

### Noncanonical and canonical activities of viral RNRs

**Fig. 6.**
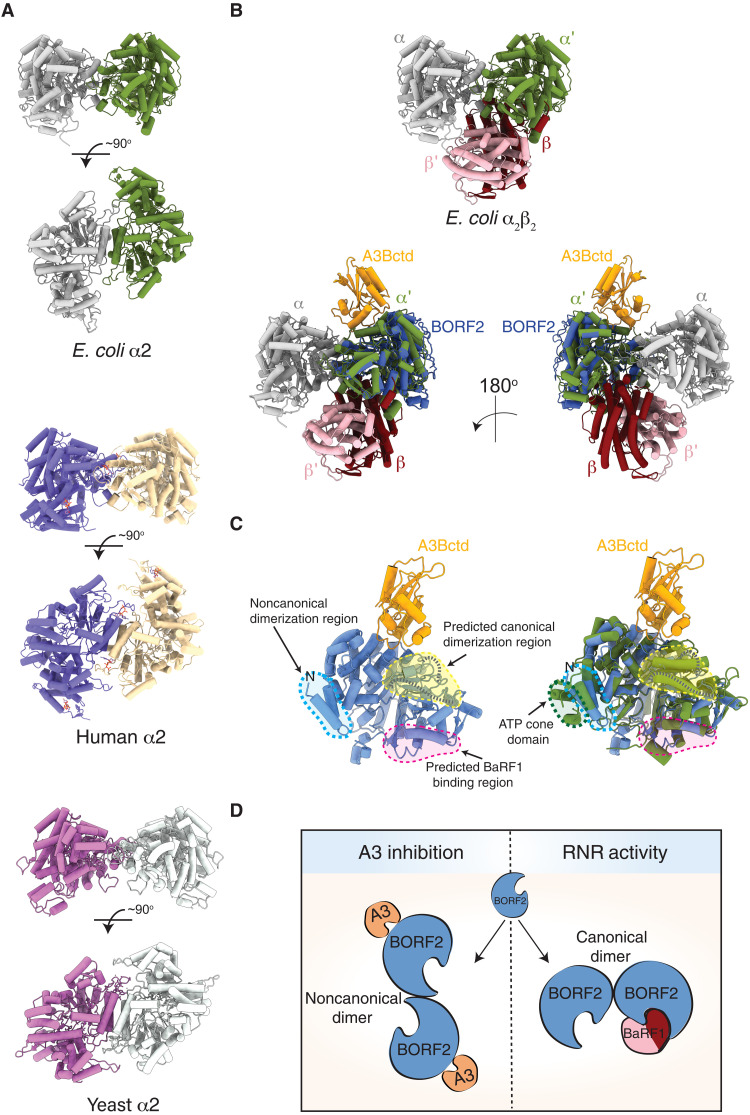
Models for BORF2 function in ribonucleotide reduction and A3B inhibition. (**A**) Ribbon schematics of *E. coli* (PDB: 6w4x), human (PDB: 6aui), and yeast (PDB: 2eud) canonical RNR α subunit dimers. (**B**) Top: Ribbon schematic of *E. coli* α2/β2 structure (PDB: 6w4x). Bottom: Overlay of the *E. coli* structure with a single BORF2-A3Bctd complex. The predicted BaRF1 binding surface is likely to be similar to the positioning of the *E. coli* β subunit and opposite that of the observed A3B binding surface. (**C**) Left: Ribbon schematic of BORF2-A3Bctd monomer, with a yellow circle showing the noncanonical BORF2 dimerization region and a red circle highlighting the predicted canonical dimerization domain. Blue dashed lines represent BORF2 disordered regions. Right: Identical to the left schematic, except showing an additional overlay of the *E. coli* α subunit in green (PDB: 6w4x). (**D**) Cartoon schematics of the two different BORF2 dimerization mechanisms, the noncanonical dimer observed here for A3B inhibition (left) and the canonical dimer predicted to be required for ribonucleotide reduction (right).

The canonical dimer formed between large subunits of class Ia RNRs is conserved and required for ribonucleotide reduction (e.g., schematics of *E. coli* and human complexes in Fig. 6A) (*1*, *6*). EBV carries genes for both the large RNR subunit, BORF2, and the small subunit, BaRF1, and both are expressed during the lytic stage of virus replication (*33*–*35*). A recent cryo-EM structure of an *E. coli* RNR complex trapped in an active state provided unprecedented detail for the RNR catalytic mechanism that involves orchestrated radical transfer between the small subunit (β) and large subunit (α) (Figs. 2, B and C, and 6, A and B, and fig. S8B) (*36*). A structural overlay of this *E. coli* RNR α_2_β_2_ complex and a monomer of the BORF2-A3Bctd complex shows that the A3Bctd binding site is on the opposite side of the protein and is unlikely to overlap with the predicted BaRF1 interaction region (Fig. 6, B to D). Moreover, although the structure of full-length A3B has yet to be determined, the N-terminal end of the catalytic domain is positioned away from the BORF2-A3Bctd complex (fig. S11).

These structures and inferences, together with the fact that the predicted BORF2 catalytic residues are highly conserved with the analogous *E. coli* RNR residues (Fig. 2, B and C), suggest that the BORF2-BaRF1 interaction will be positioned in a similar fashion to the *E. coli* large and small subunits, respectively (Fig. 6, B and C). RNR activity may therefore require the formation of a canonical dimer similar to other class Ia RNRs (depicted in Fig. 6C). This predicted canonical dimerization region is disordered in the BORF2-A3Bctd structure described here but predicted by AlphaFold to form a class Ia–like helical region (fig. S12). Given the flexibility detected in the BORF2-A3Bctd complex (movies S1 and S2) and the fact that other large RNR subunits show dynamic oligomerization profiles depending on the activity state of the RNR [reviewed in (*1*, *6*)], it is possible that this region may become better ordered upon canonical dimer formation. Together, we propose that BORF2 is likely to exist in at least two different dimeric complexes in cells: a noncanonical dimer shown here to bind A3Bctd and a canonical dimer to elevate cellular deoxynucleotide triphosphate (dNTP) levels (schematic in Fig. 6D).

## DISCUSSION

Here, we report a cryo-EM structure of the large RNR subunit of EBV, BORF2, in complex with the A3B catalytic domain. Structural comparisons with *E. coli*, yeast, and human class Ia large subunits show an archetypal α/β barrel RNR core and a superposition of residues required for catalysis. However, EBV BORF2 appears to lack an analogous N-terminal ATP cone domain, which is required for sensing cellular nucleoside levels and regulating ribonucleotide reduction activity [reviewed in (*1*, *4*, *32*)]. This suggests that the BORF2-BaRF1 complex may be constitutively active during EBV lytic DNA replication. Answering this question will also require addressing whether BORF2 has the capacity to form canonical dimers like other class Ia RNRs and determining how it binds to the small RNR subunit, BaRF1, to accomplish catalysis. Together, we postulate that BORF2 functions in two distinct complexes in cells, a BORF2-BaRF1 complex to boost nuclear dNTP concentrations and a BORF2-A3B complex to protect the integrity of viral genomic DNA during replication (Fig. 6D).

The cryo-EM structure also reveals a novel BORF2 dimerization mechanism (Fig. 5). This noncanonical dimer interface is composed of a four-helix bundle with two α helices from each monomer. Single charge-changing amino acid substitutions at this interface partly or fully prevent the formation of large BORF2-A3B cytoplasmic aggregates. Compensatory charge changes in which one monomer has a new negative charge and the other has a new positive charge restore the accumulation of large wild type–like cytoplasmic aggregates. The same single amino acid substitution mutant also shows a uniformly slower SEC elution profile in a manner that is fully restored by the double mutant. These results provide strong evidence that the noncanonical dimer interface observed in the cryo-EM structure is required for the formation of BORF2-A3B cytoplasmic aggregates. Large cytoplasmic aggregates have also been reported for A3s and the RNR large subunits of related herpesviruses KSHV and HSV-1, suggesting mechanistic conservation (*12*). Given the distinct nature of the oblong cytoplasmic aggregates in fluorescence microscopy images, it is likely that additional surfaces may be involved to promote higher-order oligomerization. However, it is also important to emphasize that this noncanonical dimerization activity is dispensable for the EBV large RNR subunit BORF2 to bind and relocalize A3B from its normal nuclear location into the cytoplasmic compartment of cells.

The BORF2-A3B interface is >1000 Å^2^ and comprises multiple structural elements from each protein. In particular, L1 and L7 of A3B are sequestered by multiple interactions with BORF2 including contacts with the novel short-helix insertion (SHI) and with an additional helical loop structure involving residues R484 and Y481. The net result is a high-affinity interaction that effectively blocks the A3B active site from binding to single-stranded DNA and catalyzing deamination. EBV BORF2 selectively binds to A3B over related human A3 family members (Fig. 4). Even A3A, which has the highest overall identity with the catalytic domain of A3B (>90%) and an identical L7 region, is a less preferred binding partner due to amino acid differences in L1. The strong preference of BORF2 for A3B may have been an evolutionary adaptation necessary for EBV to replicate in the nuclear compartment of human cell types such as B lymphocytes (*37*), which are capable of expressing A3B (*38*, *39*). Binding and relocalization experiments combined to demonstrate that the A3Bctd L7 region is the most critical determinant of this direct host-pathogen interaction. In comparison, the A3Bctd L1 region likely serves to help strengthen the interaction. This is evidenced by reciprocal L1 phenotypes in co-IP experiments and no overt changes in fluorescence microscopy experiments. This may be due to compensatory avidity interactions during cytoplasmic aggregate formation in living cells and/or to other as-yet-unidentified surfaces and cellular factors that somehow promote aggregation.

Viruses that are susceptible to restriction by cellular A3s have evolved potent counter-defense mechanisms [reviewed in (*5*, *15*, *40*, *41*)]. HIV-1 and related lentiviruses dedicate an accessory protein, Vif, to nucleate the formation of an E3 ubiquitin ligase complex to selectively degrade up to five different cytoplasmic APOBEC3s. Other reverse-transcribing viruses appear to use evasion [human T cell leukemia virus–1 (HTLV-1)] or sequestration (foamy virus) strategies to escape restriction, but the mechanisms are not as well worked out. The A3B neutralization mechanism of EBV BORF2 is unique, as it is the first for a double-stranded DNA virus and the first to involve a direct inhibition of DNA deaminase activity. The cryo-EM structure described here therefore suggests a strategy for A3B inhibition, which may be useful for suppressing tumor evolution potentially driven by this enzyme [reviewed in (*42*, *43*)]. For instance, a mimic of the BORF2 Y481 region may be a starting point for development of an effective A3B inhibitor. On the flip side, strategies to disrupt the BORF2-A3B interaction, such as competitive peptidomimetics of A3B L1 and/or L7, may restore A3B’s natural ability to restrict the replication of EBV and potentially also related herpesviruses and thus contribute to treating lytic phase diseases such as infectious mononucleosis, cold sores, and genital lesions.

## MATERIALS AND METHODS

### Bacterial and human cell expression constructs

*E. coli* expression vectors with an N-terminal MBP tag or 6x-HisSumo tag and a codon-optimized BORF2 gene were gifts from H. Aihara. To create the MBP-BORF2 (1 to 826) human cell expression vector, the MBP-codon–optimized BORF2 was polymerase chain reaction (PCR)–amplified from the bacterial expression constructs above using primers 5′-AAGCTTGGTACCACCATGAAAATCGAAGAAGGTAAACTGGTAATCTGGATTAACG and 5′-CTAGACTCGAGTCACTGGCAGCTTTCGCACGCGCGTTCAG and ligated into pcDNA3.1 (Invitrogen) using restriction enzymes Kpn I–HF (NEB, #R3142) and Xho I (NEB, #R0146). This construct was used to express protein for determining the cryo-EM structure, BLI experiments in Fig. 4E, and co-IP experiments in Fig. 5C. The MBP-BORF2 D26R, R39E, R39D, R39E/D26R, and R39D/D26R substitutions were made by site-directed mutagenesis. These constructs were used for co-IP experiments in Fig. 6C and fig. S10.

The N-terminal–tagged mCherry and C-terminal 3xFLAG–tagged BORF2 used in localization and co-IP studies were cloned into pcDNA5TO and pcDNA4TO (Invitrogen). The BORF2 starting methionine in the mCherry-BORF2 construct is deleted to prevent internal translation initiation. All mCherry-BORF2 and BORF2-3xFLAG single-point amino acid substitutions were generated by site-directed mutagenesis.

The A3-mycHis constructs used to express protein for structural work, biochemical assays, and BLI assays have been described (*44*). Briefly, A3Bctd (193–382)–mycHis, A3A (1–199)–mycHis, and A3G (1–384)–mycHis constructs are in pcDNA3.1 C-terminal mycHis vectors (Invitrogen). The A3A and A3B coding sequence is disrupted by an intron to avoid toxicity in *E. coli* during cloning and plasmid preparation.

Full-length A3B-eGFP, A3Bctd_193–382_-eGFP, full-length A3A-eGFP, and A3Gctd_197–384_-eGFP used in localization experiments (Figs. 4 and 5 and figs. S7 and S9) and co-IP experiments (Figs. 3 and 4 and fig. S6) were cloned into pCDNA5TO (Invitrogen) using PCR amplification, restriction enzyme digestion, and ligation. The loop swap constructs A3Bctd_193–382_A3AL1_25–30_, A3Bctd_193–382_L1_deltaPLV_, A3Bctd_193–382_A3GL1_209–217_, A3A_A3BL1_205–213_, A3A_A3GL1_209–213_, A3A_A3GL3_247–254_, A3Gctd_197–384_A3BL7_315–320,_ and A3Gctd_197–384_A3AL1_25–30_ were made by site-directed mutagenesis.

Unless otherwise stated, PfuUltra II was used for site-directed mutagenesis and HF-Phusion (NEB, #M0530) for all other PCRs. All constructs were sequence-confirmed using Sanger sequencing. Wild-type constructs match the following GenBank accessions: BORF2 (V01555.2), A3B (NM_004900), A3A (NM_145699), and A3G (NM_021822).

### Protein expression and purification

Full-length BORF2 was expressed in 293T cells as an N-terminal MBP–tagged fusion. 293T cells were grown at 37°C CO_2_ in RPMI media supplemented with 10% fetal bovine serum and 1× antibiotic-antimycotic solution (Gibco Life Technologies). Between 10 and 30 15-cm^3^ plates of 293Ts were transfected with 10 to 20 μg of plasmid using a 3:1 ratio of PEI (Polyethylenimine) to plasmid DNA. The next day, fresh medium was exchanged and the cells were incubated for an additional 36 to 48 hours. Cells were harvested and frozen at −80°C and thawed and resuspended in lysis buffer [50 mM tris-HCl (pH 7.4), 300 mM NaCl, 10% glycerol, 1 tablet of EDTA-free protease inhibitor, and ribonuclease (RNase) A (20 μg/ml)]. The resuspension was sonicated on an ice bath two to three times using a Branson sonifier (duty cycle, 4; output, 4; 2-min pulses). Cellular debris was removed by centrifugation at 15,000*g* for 45 min at 12°C. MBP-BORF2 was purified by binding to amylose resin (NEB, #E8021S). Resin was washed with 50 mM tris-HCl, 300 mM NaCl, and 10% glycerol, and MBP-BORF2 was eluted from the resin with 100 mM maltose. TCEP (final concentration, 1 mM; Thermo Fisher Scientific, #77720) was added to the elution, and the protein was concentrated to ~0.5 ml. The sample was injected into an S200 Increase 30/300 column (GE Healthcare) preequilibrated with 20 mM tris-HCl (pH 8) and 150 mM NaCl. Purified complex corresponding to the peak fraction was collected, TCEP was added (1 mM), and the protein was concentrated before experimentation.

A3Bctd-mycHis and A3A-mycHis proteins were expressed by transfecting 10 to 30 15-cm^3^ plates of 293Ts with up to 10 μg of plasmid using a ratio of 3:1 PEI to plasmid DNA. The cells were harvested ~48 hours after transfection. Pellets were frozen at −80°C. The pellets were thawed and resuspended in 50 mM tris-HCl (pH 7.4 or 8), 500 mM NaCl, 10% glycerol, 5 mM imidazole, 1 tablet of EDTA-free protease inhibitor, and RNase A (20 μg/ml). Cells were lysed in an ice bath by sonication using a Branson sonifier (duty cycle, 4; output, 4; 2-min pulses; two to three times) followed by centrifugation (15,000*g*, 45 min, 12°C). Supernatants were applied to Talon metal affinity resin (Clontech Takara, #635503) to capture the His-tagged proteins. The resin was washed with 50 mM tris-HCl (pH 8), 500 mM NaCl, and 5 mM imidazole. Bound proteins were eluted with 50 mM tris-HCl (pH 7.4 or 8), 500 mM NaCl, and 250 mM imidazole. TCEP was added to the elution (final concentration, 1 mM), and the sample was concentrated to 0.5 ml. The sample was further purified by SEC using an S200 Increase 30/300 column (GE Healthcare/Cytiva) that was preequilibrated with 20 mM tris-HCl (pH 8) and 300 mM NaCl. A3G-mycHis was purified similarly except with an additional RNase A treatment before SEC.

The MBP-BORF2/A3B-mycHis complex used to determine the cryo-EM structure was purified by mixing excess purified A3B-mycHis with MBP-BORF2 (after amylose resin purification). The complex was isolated by SEC in buffer containing 20 mM tris-HCl (pH 8) and 150 mM NaCl. TCEP was added to a final concentration of 1 mM, and the complex was concentrated and stored at −80°C.

Protein concentrations were measured by UV280 absorbance using the following molecular weights and extinction coefficients estimated using the Expasy ProtParam online tool: A3Bctd-mycHis (25,313 Da, 47,900 M^−1^ cm^−1^), A3G-mycHis (49,298 Da, 106,800 M^−1^ cm^−1^), A3A-mycHis (25,672 Da, 39,420 M^−1^ cm^−1^), and MBP-BORF2 monomer (133,261 Da, 183,010 M^−1^ cm^−1^).

### Size exclusion analyses

For the SEC profiles shown in Fig. 5B, MBP-BORF2 and mutant derivatives were expressed and purified with amylose resin as described above. Approximately 150 μg of each protein was applied to an S200 Increase 30/300 (Cytiva) size exclusion column equilibrated in 10 mM tris-HCl (pH 8) and 150 mM NaCl.

### DNA deaminase activity assays

Purified A3Bctd-mycHis was diluted to 100 nM in reaction buffer containing 10 mM Hepes (pH 7.4), 5 mM EDTA, 50 mM NaCl, and 0.5 mM. MBP-BORF2 was serially diluted in the same buffer to concentrations of 200, 100, and 50 nM. Equal volumes of MBP-BORF2 and A3Bctd-mycHis were mixed together before adding oligonucleotide. The oligonucleotide with a 3′ FAM used as a substrate for A3Bctd was ordered from IDT and contains the preferred A3B trinucleotide sequence “TCA” (ATTATTATTATTCAAATGGATTTATTTATTTATTTATTTATTT-FAM). The oligonucleotide was diluted to 800 nM in reaction buffer. Equal volumes of substrate and protein complex were mixed together and incubated for 1 hour at 37°C (total reaction volume, 10 μl). This was followed by addition of UDG (Uracil-DNA Glycosylase) (NEB, M0280L) for 10 min to remove the uracil base resulting from A3B deaminase activity. The reaction mix was then incubated with 1 M NaOH for 10 min at 98°C to break the phosphodiester backbone at the abasic site. Loading dye (11 μl) was added to each reaction, and reaction products were separated on a 15% tris-borate EDTA–urea acrylamide gel. The gel was imaged using Typhoon 7000 (GE Healthcare).

### BLI assays

BLI assays were performed using Octet RED (FortéBio). A3Bctd-mycHis, A3A-mycHis, and full-length A3G-mycHis were loaded onto Ni–nitrilotriacetic acid biosensors (FortéBio, catalog no. 185101) at 12.5 μg/ml for A3Bctd and A3A and 8 μg/ml for A3G. MBP-BORF2 was serially diluted (200, 100, 50, 25, 12.5, and 6.25 nM). All proteins were diluted in buffer containing 20 mM tris-HCl (pH 8) and 300 mM NaCl (reaction buffer). The assay was performed in 384-well tilted-bottom microplates (FortéBio, catalog no. 18-5076). Reaction buffer (60-μl volumes) or protein samples (60-μl volumes) were used in each condition. Before running the assay, the biosensor probes were hydrated in reaction buffer for at least 10 min. The assay was set up in the following order: (i) equilibration in buffer, (ii) loading of A3-mycHis proteins (300 s), (iii) washing/equilibration in buffer, (iv) association with BORF2 (1200 s), and (v) dissociation in buffer (1800 s). The reaction temperature was set at 30°C. The results were analyzed using FortéBio Octet data analysis software 11.1.2. Wells containing no BORF2 were assigned as the reference sample. The data best fit a 1:2 binding model.

### IF microscopy experiments

HeLa cells grown in RPMI media were transduced with a pLENTI6 TetRepressor construct and selected with blasticidin (5 μg/ml; GoldBio, #B-800-25). The cells were subcloned by serial dilution to obtain single-cell clones. The clonal populations were stably transfected with pcDNA5TO vectors containing eGFP, A3Bctd-eGFP, or A3B-eGFP. The cells were selected with hygromycin (GoldBio, #H-270-1) and subcloned by serial dilution. Representative clones were used for IF experiments (Fig. 5D and figs. S9 and S10).

Approximately 3000 engineered HeLa cells were plated into each well of a 96-well imaging plate (Coning, #3904). The next day, cells were transfected with 25 ng of pcDNA5TO-mCherry-BORF2 or single/double amino acid substitution mutant derivatives using TransIT-LTI (Mirus, #MIR 2300). The cells were treated with doxycycline (50 ng/μl) approximately 24 hours later. The following day, cells were washed with 1× phosphate-buffered saline and fixed using 4% formaldehyde (Thermo Fisher Scientific, #28906), and the nuclei were stained with Hoechst 33342 (Thermo Fisher Scientific, #62249). For IF experiments shown in Fig. 4F and fig. S7, parental (non-engineered) HeLa cells were transfected transiently with 25 ng of pcDNA4TO-BORF2-FLAG and 25 ng of pcDNA5TO-A3-eGFP constructs using TransIT-LT1 (Mirus, #MIR 2300). The following day, cells were fixed using 4% formaldehyde (Thermo Fisher Scientific, #28906), permeabilized in 0.2% Triton X-100 (Sigma-Aldrich, #T8787) for 10 min, and incubated in blocking buffer [2.8 mM KH_2_PO_4_, 7.2 mM K_2_HPO_4_, 5% goat serum (Gibco, #16210064), 5% glycerol, 1% cold water fish gelatin (Sigma-Aldrich, #G7041), and 0.04% sodium azide (pH 7.2)] for 1 hour. Cells were then incubated with primary antibody mouse anti-FLAG (Sigma-Aldrich, #F1804, 1:1000) overnight at 4°C to detect FLAG-tagged BORF2. The next day, cells were incubated with the secondary antibody Alexa Fluor 594 (Invitrogen, #A-11005, 1:1000) for 2 hours at room temperature protected from light, and nuclei were stained with Hoechst 33342 (Thermo Fisher Scientific, #62249). Images were collected using Cytation 1 (BioTek). A total of 50 images per well were collected at ×20 magnification using the automated imaging function. Background subtraction was performed using Fiji (*45*), and images were analyzed using CellProfiler v4.2 (*46*). A pipeline was generated to trace cells, segment the cytoplasmic and nuclear compartments, and identify aggregates/oblong structures in the cytoplasm. Images showing cell segmentation of nuclear and cytoplasmic compartments (middle; yellow and cyan tracing) and aggregate and oblong structures (right; pink tracing) are shown in fig. S13. At least 50 cells were quantified for each condition. CellProfiler Analyst v3.0.1 (*47*) was used for scoring phenotypes by machine learning to quantify the population of cells within each condition with relocalized A3Bctd-eGFP, full-length A3B-eGFP, eGFP, and/or aggregates/oblong structures (Figs. 4F and 5D and figs. S9 and S10).

### Immunoprecipitation experiments

Immunoprecipitation experiments in Figs. 3 and 4 and fig. S6 were performed as described (*11*) with a few adjustments. Briefly, 250,000 293T cells in six-well plates were transfected with 100 ng of each construct using TransIT-LTI (Mirus, #MIR 2304). Cells were harvested ~48 hours after transfection and resuspended in 700 μl of lysis buffer [50 mM tris-HCl (pH 7.5), 150 mM NaCl, 10% glycerol, 0.5% Tergitol, 1 of tablet EDTA-free protease inhibitor, and RNase A (100 μg/ml)]. Resuspension was sonicated on ice bath for 10 s at the lowest setting using a Branson sonifier and then centrifuged at 15,000 rpm for 10 min. A 30-μl aliquot of supernatant was removed for input detection. The remaining supernatant was incubated with 25 μl of resin anti-FLAG magnetic beads (Sigma-Aldrich, #M8823) per sample overnight with gentle rotation at 4°C. Samples were washed three times with lysis buffer and eluted with 30 μl of elution buffer [50 mM tris-HCl (pH 7.5), 150 mM NaCl, 0.05% Tergitol, 10% glycerol, and FLAG peptide (100 μg/ml)]. The immunoprecipitations described in Fig. 5 were done using the same protocol except that 400,000 293T cells were transfected with 200 ng of each construct.

Elution and input for assays described in Figs. 3 and 4 and fig. S6 were evaluated by immunoblotting with rabbit anti-FLAG at 1:5000 to detect BORF2-3xFLAG (Sigma-Aldrich, #F7425), mouse anti-GFP at 1:5000 dilution to detect A3-GFP (Living Colors, #JL-8), and rabbit anti-actin at 1:5000 (Cell Signaling Technology, #13E5) to detect actin (loading control). Secondary antibodies used were IRDye 680RD anti-rabbit (LI-COR, #925-68071) and horseradish peroxidase–linked anti-mouse (Cell Signaling Technology, #7076), both at 1:10,000 dilution. Elutions and inputs described in Fig. 5 were detected by immunoblot using mouse anti-FLAG at 1:4000 dilution, rabbit anti-MBP (Invitrogen, #PA1.989) at 1:3000 dilution, and rabbit anti-actin listed above at 1:5000 dilution. Secondary antibodies used were IRDye 680RD anti-rabbit (LI-COR, #925-68071) and IRDye 800CW anti-mouse (LI-COR, #926-32210) at 1:20,000 dilution.

### Cryo-EM grid preparation

Quantifoil grids R-1.2/1.3 Cu 400 mesh (Electron Microscopy Sciences) were glow-discharged for 1 min at 15 mA using a PELCO easiGlow apparatus. Sample (3 μl) at 0.33 mg/ml was applied to the grid, and FEI Vitrobot Mark IV was used to plunge-freeze grids with the following parameters (temperature, 6°C; humidity, 95%; blotting force, 3; blotting time, 3). After blotting, the grid was rapidly frozen in liquid ethane.

### Cryo-EM data collection

A total of 7488 movies were collected from a frozen grid imaged on a Titan Krios cryo–electron microscope (FEI/Janelia Research Campus) operating at 300 keV and equipped with a spherical aberration corrector, an energy filter (Gatan GIF Quantum), and a post-GIF Gatan K3 direct electron detector. Movies were acquired at a calibrated magnification of ×59,242 on the K3 camera in CDS mode, corresponding to 0.844 Å per physical pixel (0.422 Å per superresolution pixel). The dose rate on the specimen was set to be 11.5 electrons/Å^2^ per second, and total exposure time was 5.22 s. Fifty frames per movie were collected at 1.2 e^−^/Å^2^ dose per frame for a total of 60 e^−^/Å^2^ dose per movie. The nominal defocus range was set at −0.8 to −2.5 μm. Camera gain reference map was taken at the start of the data collection session but not applied to each movie series to limit file size. To further save disk space, each movie series was saved in tiff format with LZW compression. Data were collected using SerialEM. Data collection parameters are shown in table S1.

### Cryo-EM data processing

Cryo-EM data were processed in cryoSPARC v3.2.0 (*20*) as depicted in the cryo-EM workflow (fig. S1). After movie alignment and CTF estimation, cryoSPARC blob picker and 2D classifications were performed to select good templates for template-based particle picking. Particles were extracted (3x-Fourier crop) followed by several rounds of 2D classification. The 2D classes showed the presence of monomeric and dimeric species of the complex. The classes corresponding to the dimeric species were selected for ab initio reconstruction and heterogeneous refinement. Particles were then reextracted without Fourier cropping followed by additional rounds of 2D classification to remove suboptimal particles. Good classes were selected for ab initio reconstruction and heterogeneous refinement, resulting in a 2.8-Å reconstruction with C2 symmetry imposed. The particles from this reconstruction were symmetry-expanded. A mask around the BORF2-A3B monomer was generated using ChimeraX (*48*), and local refinement was performed with the symmetry-expanded particle stack yielding a 2.55-Å map. A composite map from this focused refinement was generated in Phenix v1.18-3855-000 [combine focused map feature (*49*)] to represent the dimeric form of the complex. The focused refined and composite maps were sharpened using DeepEMhancer (*50*). The MBP tag on the N-terminus of BORF2 was not detected in any of the maps.

### Model building and refinement

A crystal structure of A3Bctd [Protein Data Bank (PDB): 5cqh (*44*)] and a model of BORF2 generated using Phyre2 (*51*) were docked and fitted into the cryo-EM maps using ChimeraX (*48*). The model was further corrected and built manually in Coot (*52*) and refined using real-space refinement in Phenix (*53*). Model refinement statistics are shown in table S1. All figures of the model were made using Chimera (*54*) or ChimeraX (*48*).

### Modeling using AlphaFold

The structural models depicted in fig. S4 were generated using AlphaFold (*26*). The protein model of full-length A3B depicted in fig. S11 was downloaded from the AlphaFold Structure Database (https://alphafold.ebi.ac.uk/) (*26*, *55*).

## Notes

View/request a protocol for this paper from *Bio-protocol*.
