# Supplementary material for: Cryo-EM structure of the EBV ribonucleotide reductase BORF2 and mechanism of APOBEC3B inhibition
Source: Sci Adv. 2022 Apr 27;8(17):eabm2827. doi: 10.1126/sciadv.abm2827 (PMC9045721; doi:10.1126/sciadv.abm2827)
Supplement: Supplementary file 1 — Figs. S1 to S13 Table S1 [file sciadv.abm2827_sm.pdf]

Science Advances
AAAS

Supplementary Materials for

Cryo-EM structure of the EBV ribonucleotide reductase BORF2 and mechanism of APOBEC3B inhibition

Nadine M. Shaban*, Rui Yan, Ke Shi, Sofia N. Moraes, Adam Z. Cheng, Michael A. Carpenter, Jason S. McLellan, Zhiheng Yu, Reuben S. Harris*

*Corresponding author. Email: nmshaban@umn.edu (N.M.S.); rsh@umn.edu (R.S.H.)

Published 27 April 2022, *Sci. Adv.* **8**, eabm2827 (2022)
DOI: 10.1126/sciadv.abm2827

The PDF file includes:

Figs. S1 to S13
Table S1
Legends for movies S1 and S2

Other Supplementary Material for this manuscript includes the following:

Movies S1 and S2

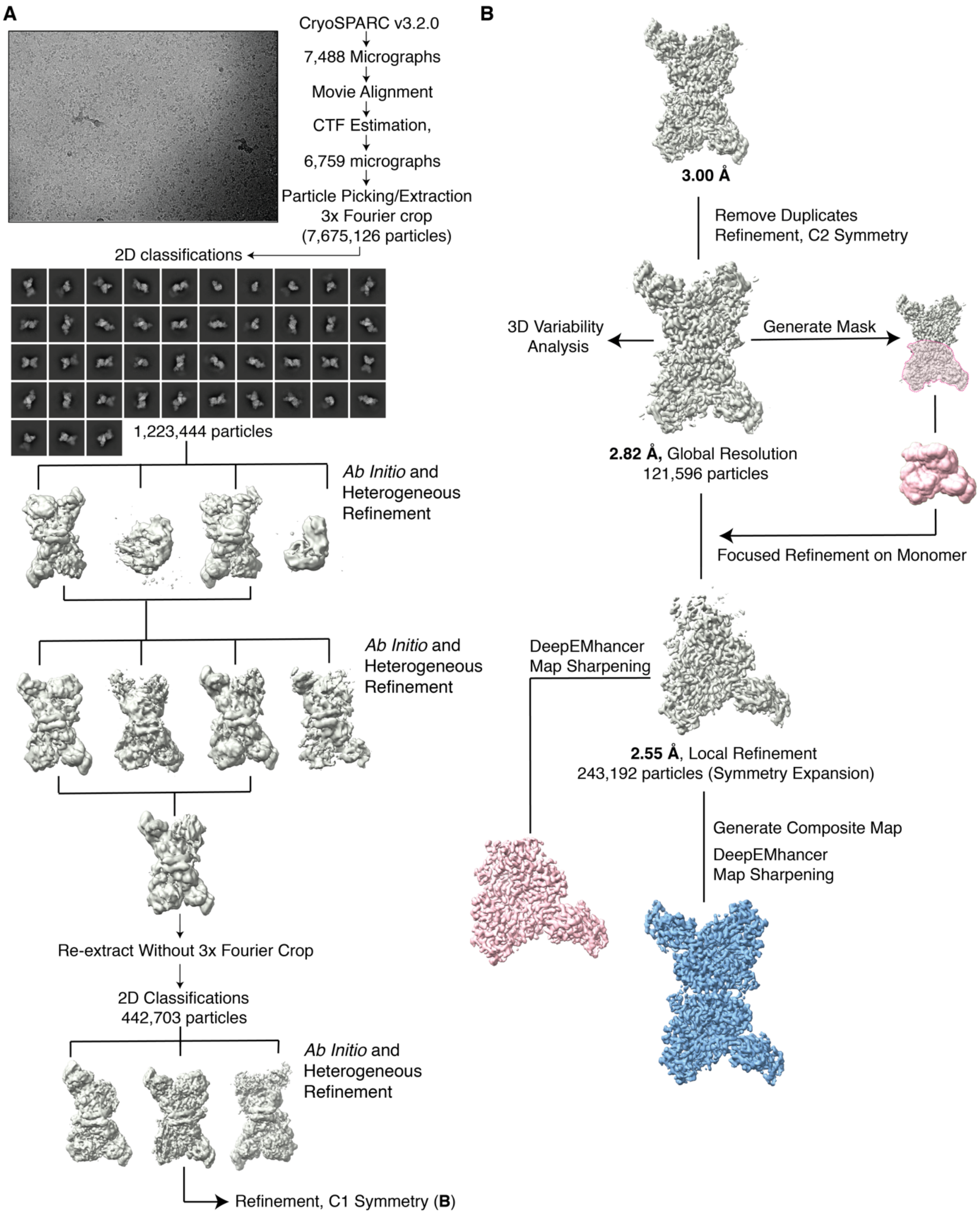

Fig. S1. Cryo-EM data processing work-flow.

(**A**) Top left: representative micrograph and 2D classes with workflow of *ab initio* reconstruction and refinement.

(**B**) Refinement workflow for reconstruction selected in panel A.

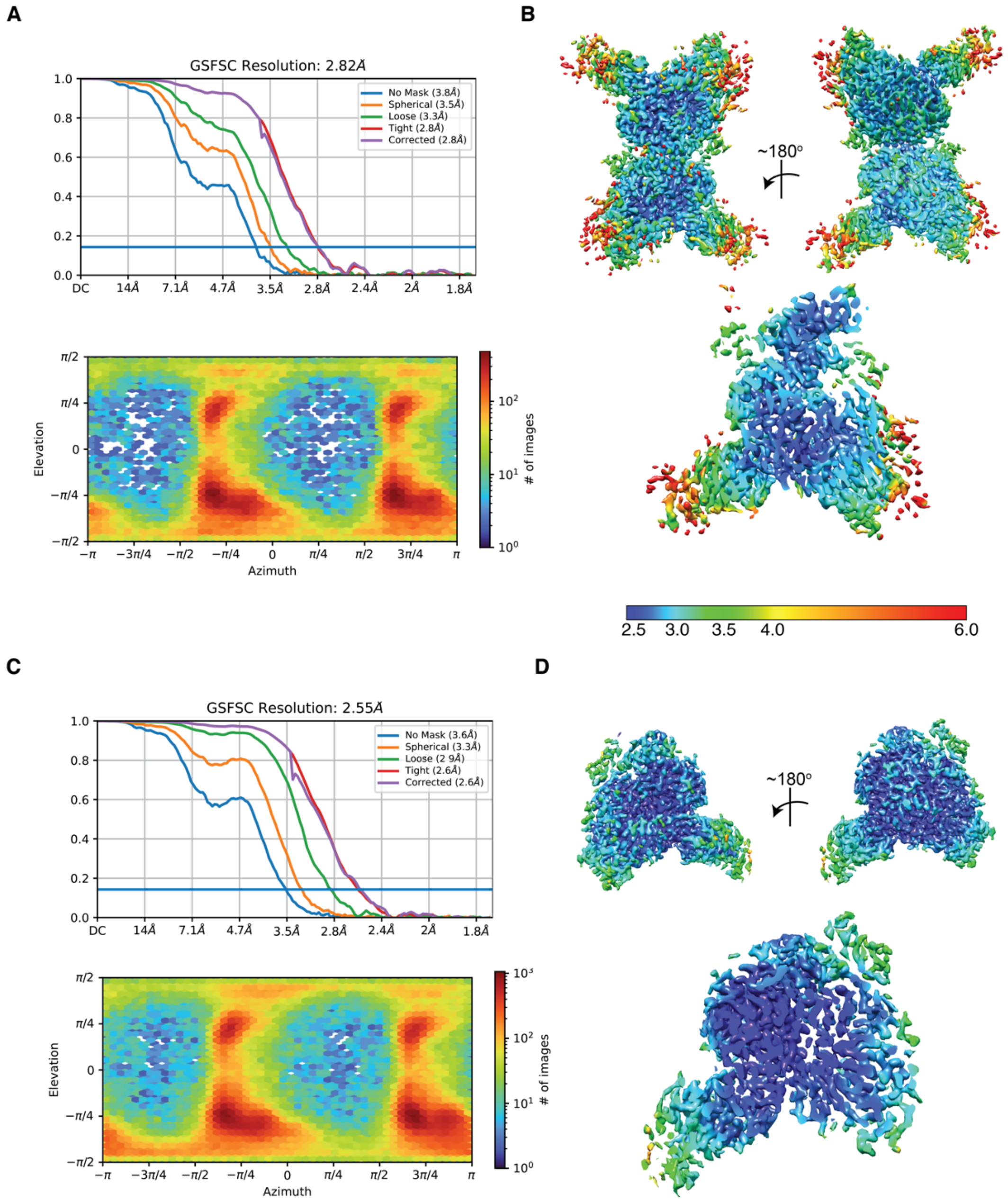

Fig. S2. Cryo-EM data validation.

(**A**) Top: FSC curves for the 2.8 Å global resolution cryo-EM reconstruction (fig. S1). Bottom: viewing direction distribution plot.

(**B**) Top: Two views of the 2.8 Å global resolution cryo-EM map colored to depict local resolution range. Bottom: close up of the BORF2-A3B monomer and central slice through one of the views showing the local resolution range. Color key shown on bottom. Resolution is in angstroms. Local resolution calculated using local estimation function in cryoSPARC.

(**C**) Top: FSC curves of 2.55 Å local refinement reconstruction (fig. S1). Bottom: viewing direction distribution plot.

(**D**) Top: Two views of the 2.55 Å local refined cryo-EM map colored to depict local resolution range. Bottom: zoom in and central slice through one of the views showing the local resolution range. Color key shown on the bottom of panel B. Resolution is in angstroms. Local resolution calculated using local estimation feature in cryoSPARC.

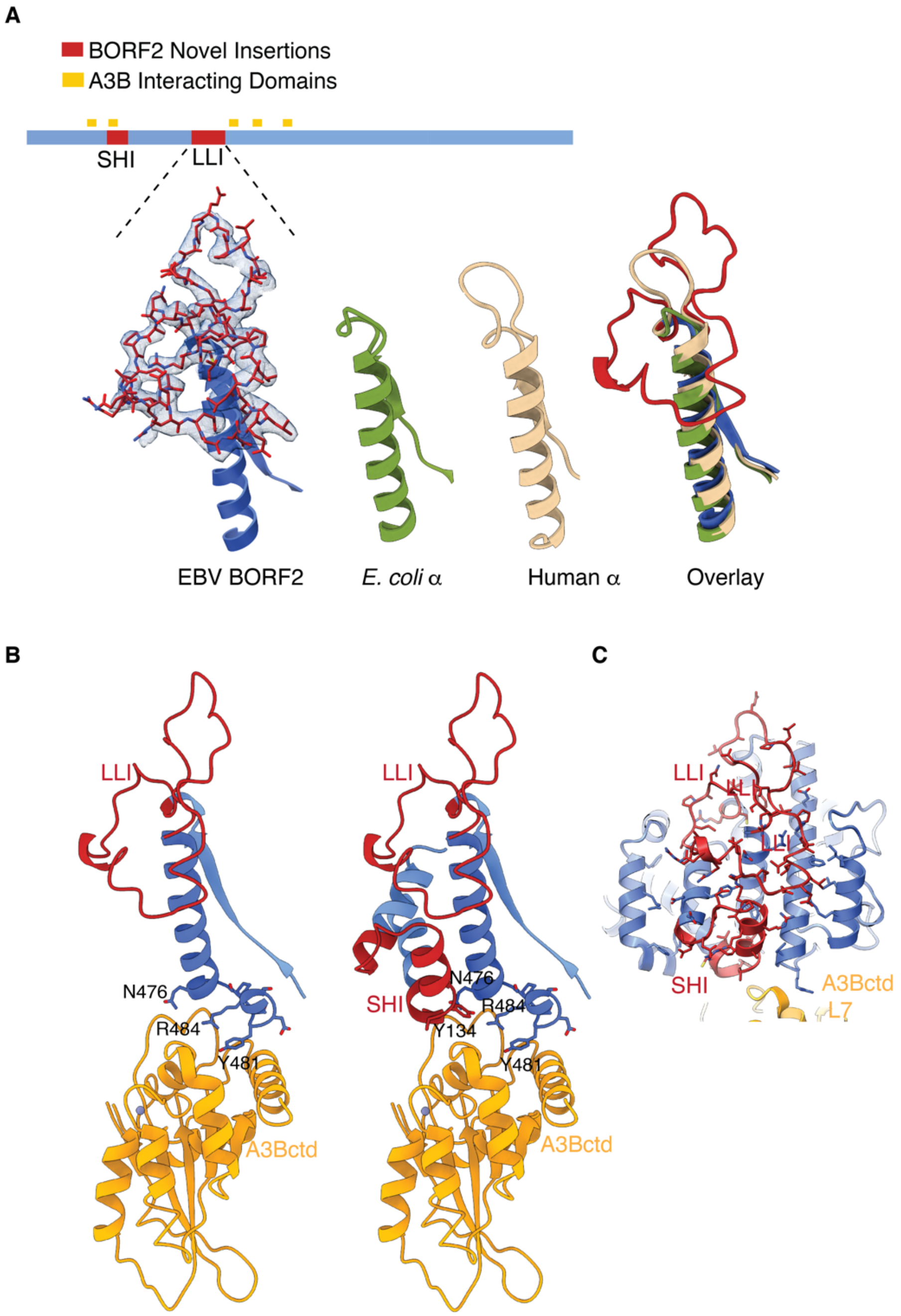

Fig. S3. Novel insertions in EBV BORF2.

(**A**) Schematic of BORF2 (1-826 amino acids) showing the novel SHI and LLI insertions (red). Select view of the BORF2 LLI insertion and the corresponding regions of the human RNR α subunit (pdb: 6aui, chain A; tan) and the *E. coli* RNR α subunit (pdb:6w4x, chain B; green). Cryo-EM map (left) for the LLI region of BORF2 (blue mesh).

(**B**) Ribbon schematics showing connectivity between BORF2 insertions LLI and SHI and A3Bctd. The left schematic emphasizes core interactions and the right schematic provides additional BORF2 structure including the SHI. BORF2 residues that interact with A3Bctd are labeled.

(**C**) A faded ribbon schematic showing the extensive interactions that occur between the SHI and LLI and also between these novel insertions and the protein core.

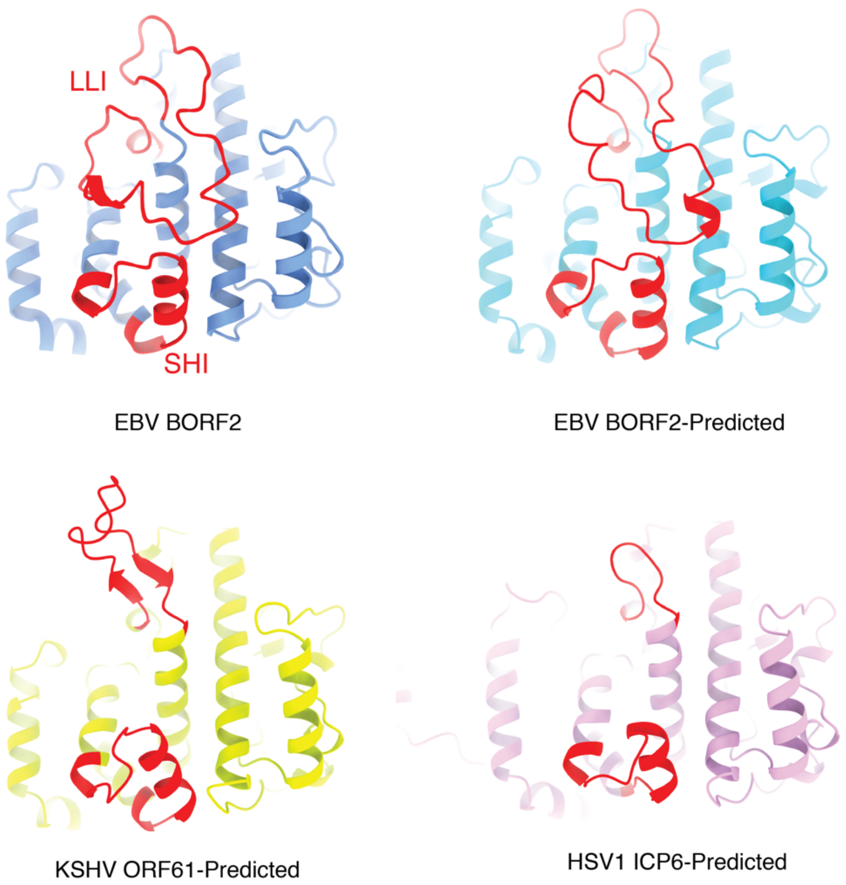


```
BORF2 EBV    VSAFAQSPAYERIVSSGYLSALRYYDTYLYV--GRSGKQESVQHFYMRLAGF----CAST  130
ORF61 KSHV   lcayrrskayedtlncgylsavrlydtyvlrtmgtepvyesvaqmfmrvsvfvacqcleh  161
ICP6 HSV1    dclihs--tphtlverglqsalkyeefylkrfg--ghymesvfqmytriagflacratrg  534
              .      : .  :. *  **:: : *:          *** ::: *:: *   .

                          SHI
BORF2 EBV    TCLYAGLRAALQRARPEIESDMEVFDYYFEHLTSQTVCCSTPFMRFAGVENSTLASCILT  190
ORF61 KSHV   eclywlardliedak--svsemaiveyvfgylaaqhvccatpilrsagveggqlascfil  219
ICP6 HSV1    mrhi---------algregswwemfkfffhrlydhqivpstpamlnlgtrnyytsscylv  585
                          *     *   :..:*  *  : :  :** :   *...  :** :

BORF2 EBV    SPFILLKEACNAHFWRDLQGEAMNAANLCAEVLQPSR-KSVATCNLANICLPRCLVNAPL  419
ORF61 KSHV   spyvllkealnehhwtdtqgmaincsnlcaeivqlpg-rntsvcnlaniclpkclrtves  447
ICP6 HSV1    spfvmfkdavnrhyiydtqgaaiagsnlcteivhpaskrssgvcnlgsvnlarcvs----  820
             **::::*:* * *.  * ** *: :***:*:::    :. ..***..: * :*:

                          LLI
BORF2 EBV    AVRAQRADTQGDELLLALPRLSVTLPGEGAVGDGFSLARLRDATQCATFVVACSILQGSP  479
ORF61 KSHV   arvgtt---dan-------------------rpffcfealgdavrvavlvinacilggsh  485
ICP6 HSV1    -------------------------------rqtfdfgrlrdavqacvlmvnimidstlq  849
                                              * :  * **.: ..:::   *
```

Fig. S4. Comparison of the large subunit of herpesviral RNRs using predictive modelling. Top: Cryo-EM structure of EBV-BORF2 in comparison to AlphaFold-predicted structures of the RNR large subunit from EBV (light blue), KSHV (yellow), and HSV-1 (pink). The actual and predicted SHI and LLI domain regions depicted in red.

Bottom: Multiple sequence alignment of two regions of EBV BORF2 (GenBank V01555.2**)**, KSHV ORF61 (GenBank QFU18774.1), and HSV-1 ICP6 (GenBank QFQ61410.1). BORF2 SHI and LLI domain regions are colored red. Sequence alignment was done using Clustal Omega multiple sequence alignment tool.

A

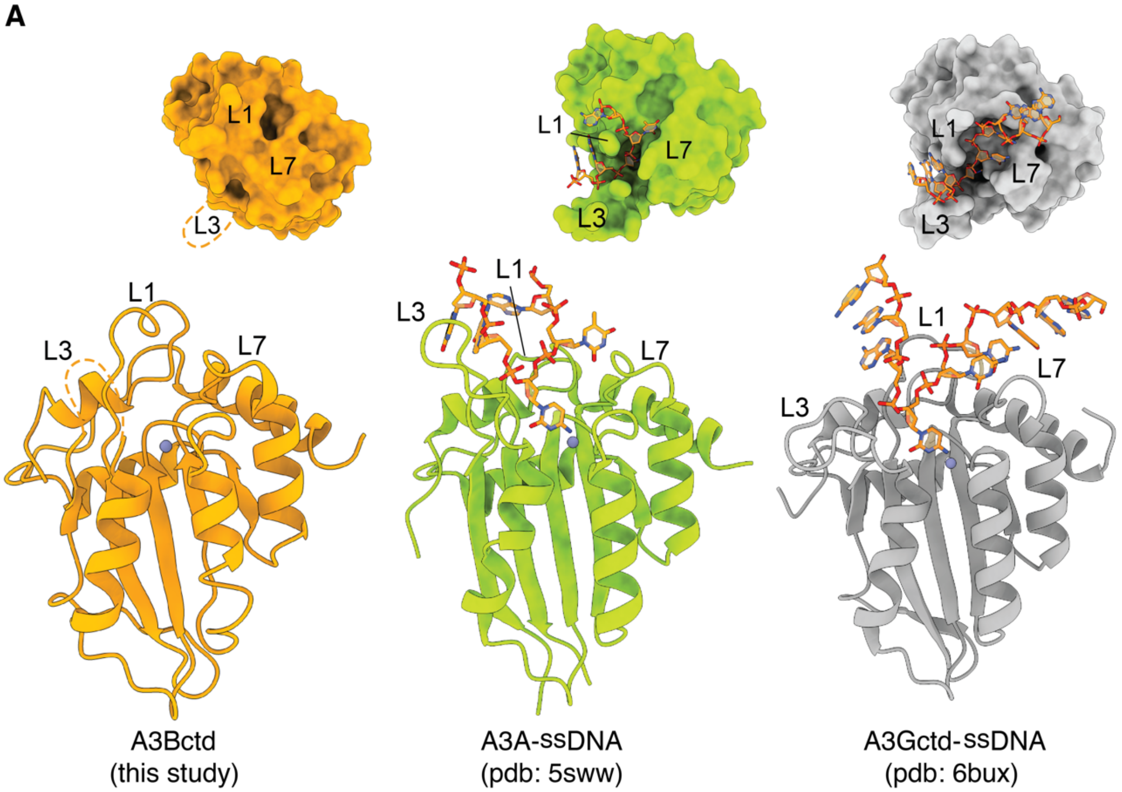

B

```
                                  L1
A3Bctd ---------RYLMDPDTFTFNFNNDPLVLRRRQTYLCYEVERLDNGTWVLMDQHMGFLCN 240
A3A    MEASPASGPRHLMDPHIFTSNFNNG---IGRHKTYLCYEVERLDNGTSVKMDQHRGFLHN 57
A3Gctd ---------RHSMDPPTFTFNFNNEPWVRGRHETYLCYEVERMHNDTWVLLNQRRGFLCN 244
                *:  ***   ** ****        *::*********:.*.*  *  ::*:  *** *

          L3
A3Bctd EAKNLLCGFYGRHAELRFLDLVPSLQLDPAQIYRVTWFISWSPCFSWGCAGEVRAFLQEN 300
A3A    QAKNLLCGFYGRHAELRFLDLVPSLQLDPAQIYRVTWFISWSPCFSWGCAGEVRAFLQEN 117
A3Gctd QAPHKHGFLEGRHAELCFLDVIPFWKLDLDQDYRVTCFTSWSPCFS--CAQEMAKFISKN 302
       :* :    : ****** ***::*  :**  * **** * *******  ** *:   *:.:*

                                L7
A3Bctd THVRLRIFAARIYDYDPLYKEALQMLRDAGAQVSIMTYDEFEYCWDTFVYRQGCPFQPWD 360
A3A    THVRLRIFAARIYDYDPLYKEALQMLRDAGAQVSIMTYDEFKHCWDTFVDHQGCPFQPWD 177
A3Gctd KHVSLCIFTARIYDDQGRCQEGLRTLAEAGAKISIMTYSEFKHCWDTFVDHQGCPFQPWD 362
       .** * **:***** :   :*.*: * :***::*****.**::****** :*********

A3Bctd GLEEHSQALSGRLRAILQNQGN 382
A3A    GLDEHSQALSGRLRAILQNQGN 199
A3Gctd GLDEHSQDLSGRLRAILQNQEN 384
       **:**** ************ *
```

Fig. S5. APOBEC3 catalytic domain structures and protein sequence alignment.
(**A**) Structures of A3Bctd (this study), A3A-ssDNA (pdb: 5sww), and A3Gctd-ssDNA (pdb: 6bux). L1, L3, and L7 regions are labelled. The space-filled representations above highlight the proximity of L1/L3/L7 to the ssDNA binding pocket.
(**B**) Protein sequence alignment of A3Bctd, A3A, and A3Gctd. Identical residues are colored with blue boxes, and the loop 1, 3, and 7 regions are highlighted in yellow. Sequence alignment was done using Clustal Omega multiple sequence alignment tool.

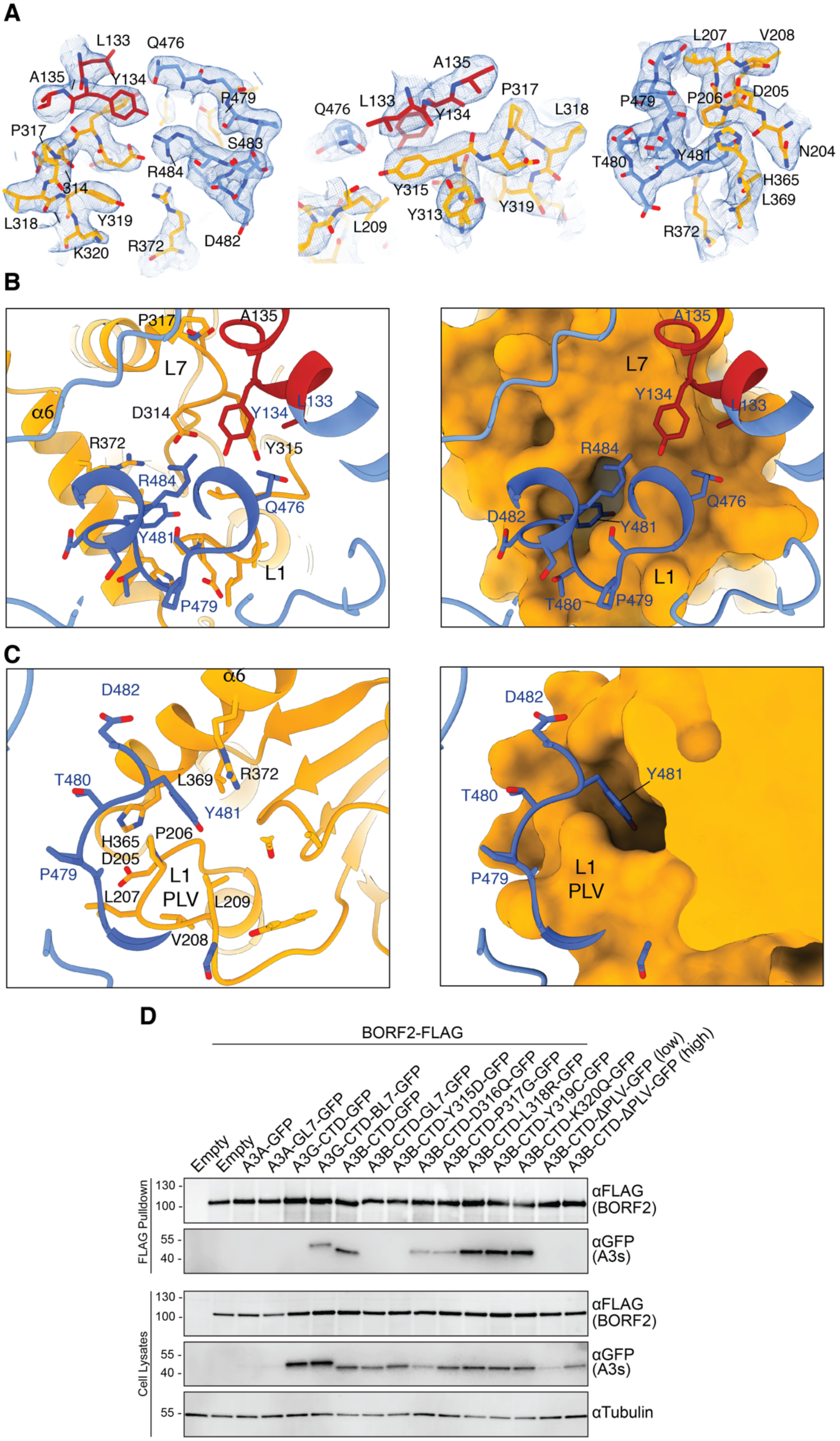

A
L133
Q476
A135
Y134
P479
P317
S483
R484
314
L318
Y319
K320
R372
D482
A135
Q476
L133
P317
Y134
L318
Y315
Y313
Y319
L209
L207
V208
P479
P206
D205
N204
T480
Y481
H365
L369
R372
B
P317
A135
L7
α6
D314
Y134
L133
R372
Y315
R484
Q476
Y481
L1
P479
A135
L7
Y134
L133
R484
D482
Q476
Y481
L1
T480
P479
C
α6
D482
T480
L369
R372
Y481
P206
H365
D205
P479
L1
PLV
L207
L209
V208
D482
Y481
T480
P479
L1
PLV
D
BORF2-FLAG
Empty
Empty
A3A-GFP
A3A-GL7-GFP
A3G-CTD-GFP
A3G-CTD-BL7-GFP
A3B-CTD-GFP
A3B-CTD-GL7-GFP
A3B-CTD-Y315D-GFP
A3B-CTD-D316Q-GFP
A3B-CTD-P317G-GFP
A3B-CTD-L318R-GFP
A3B-CTD-Y319C-GFP
A3B-CTD-K320Q-GFP
A3B-CTD-ΔPLV-GFP (low)
A3B-CTD-ΔPLV-GFP (high)
FLAG Pulldown
Cell Lysates
130
100
55
40
αFLAG
(BORF2)
αGFP
(A3s)
αTubulin

Fig. S6. Summary of key interactions between BORF2 and A3Bctd.
(**A**) Zoom-in of residues involved in interaction between BORF2 and A3Bctd with alternative views shown in the middle and right. BORF2 residues are colored blue and red, and A3Bctd residues are orange. Residues are depicted as sticks. Cryo-EM map represented by blue mesh.
(**B**) A zoom-in of the BORF2 SHI (blue/red) and A3Bctd L7 region (orange). Ribbon and stick schematic on the left, and the same view with a surface-filled representation of A3Bctd on the right.
(**C**) A zoom-in of BORF2 residue 481 (blue) and A3Bctd L1 region (orange). Ribbon and stick schematic on the left, and the same view with a surface-filled representation of A3Bctd on the right.
(**D**) BORF2 (anti-FLAG) co-IP experiments with the indicated A3-eGFP constructs including key A3Bctd mutants. The two A3A constructs were non-informative in this experiment but still included in the images here to avoid gel-cropping and allow visualization of the negative control reactions (empty +/- BORF2-FLAG). See **Fig. 3D** for additional BORF2 mutants.

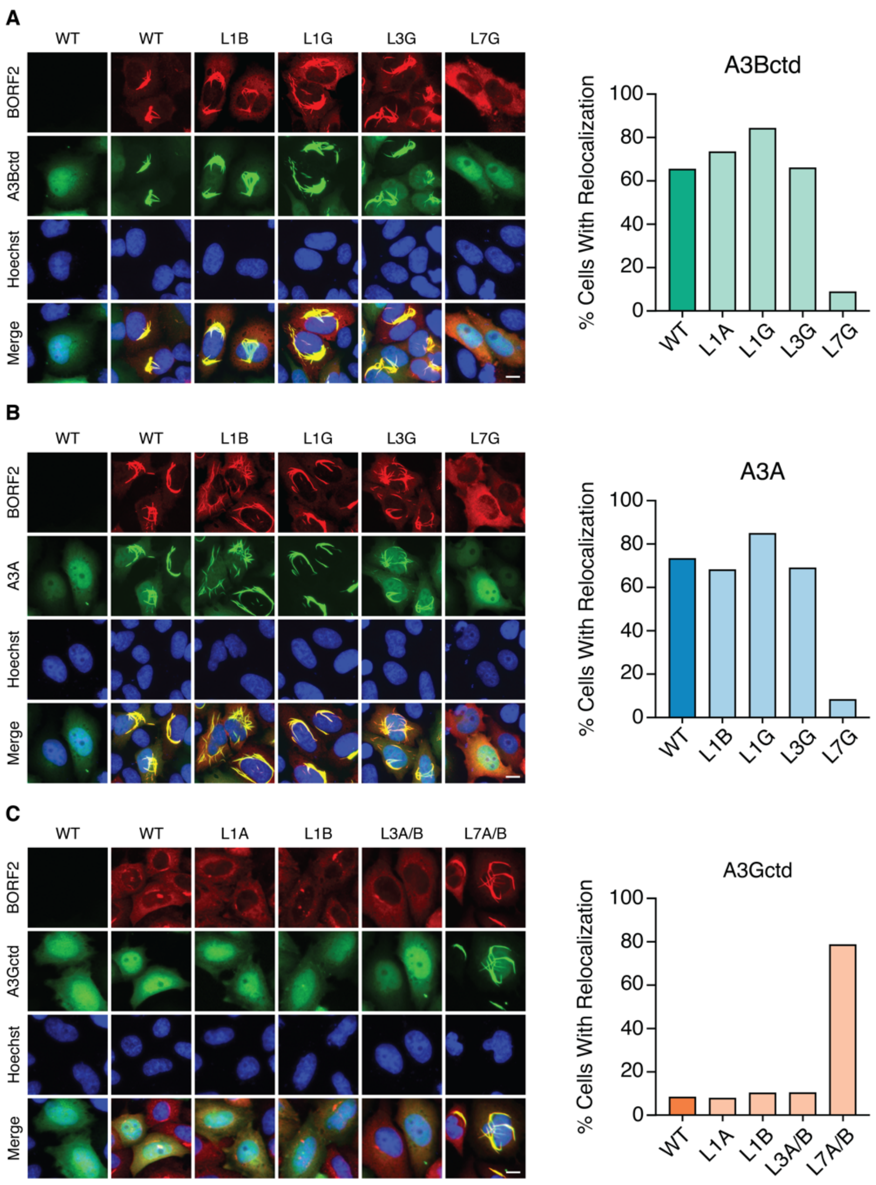

Fig. S7. Relocalization phenotypes of BORF2 and different A3 loop mutant constructs.
(**A**) Additional representative fluorescence microscopy images of BORF2-FLAG (except first image column) and the indicated A3Bctd-eGFP constructs. Nuclei are stained with Hoechst (scale = 10µm). Right – histogram showing the percentage of cells with relocalized A3Bctd-eGFP relative to non-BORF2 expressing cells (n > 50 for each condition).
(**B**) Representative fluorescence microscopy images of BORF2-FLAG (except first image column) and the indicated A3A-eGFP constructs. Nuclei are stained with Hoechst (scale = 10µm). Right – histogram showing the percentage of cells with relocalized A3A-eGFP relative to non-BORF2 expressing cells (n > 50 for each condition).
(**C**) Representative fluorescence microscopy images of BORF2-FLAG (except first image column) and the indicated A3Gctd-eGFP constructs. Nuclei are stained with Hoechst (scale = 10µm). Right – histogram showing the percentage of cells with relocalized A3Gctd-eGFP relative to non-BORF2 expressing cells (n > 50 for each condition).

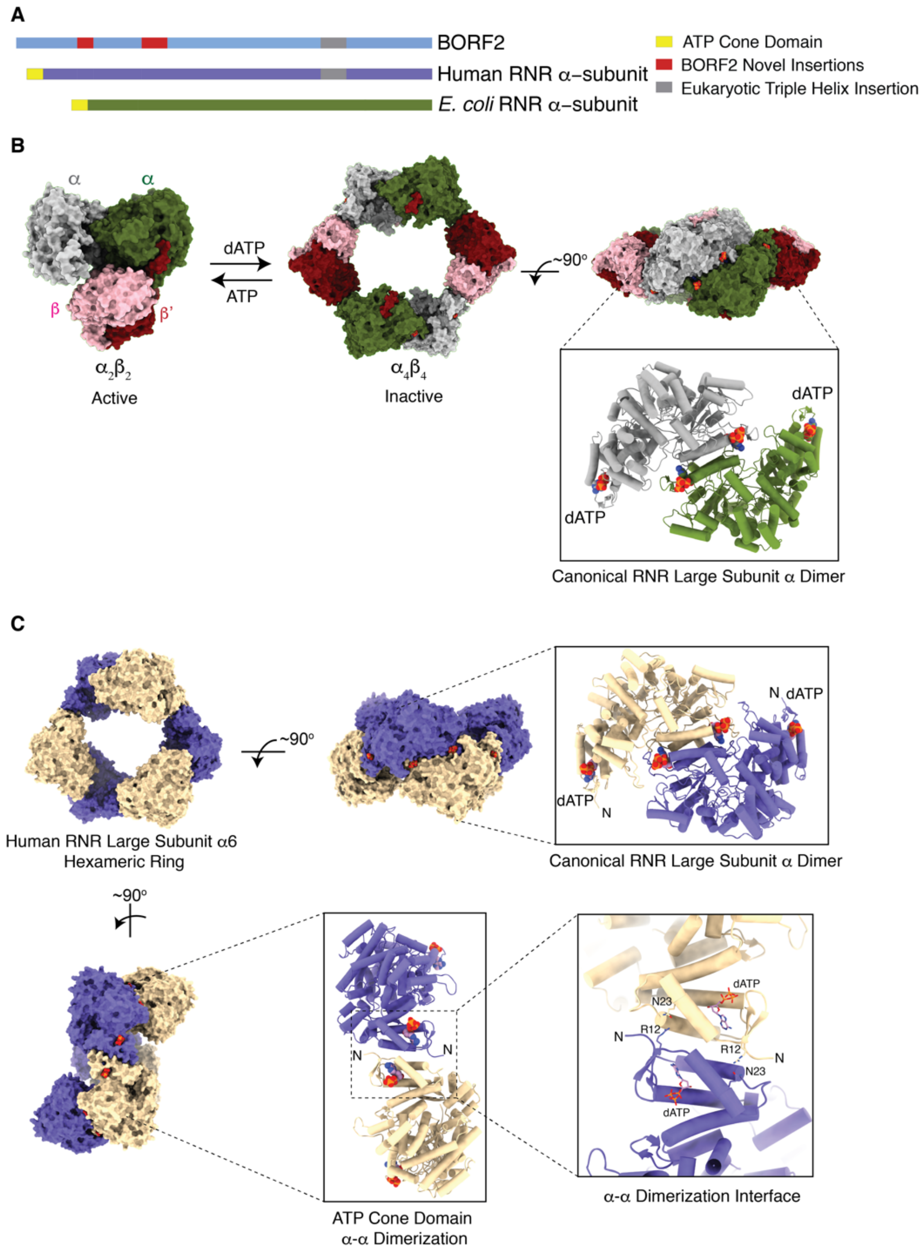

Fig. S8. N-terminal ATP cone-domain is absent in EBV BORF2 despite regulatory functions in other class1a RNRs.

(**A**) Protein schematic of EBV BORF2, human RNR α-subunit, and *E. coli* RNR α-subunit with key domains indicated.

(**B**) Surface representations of the active and inactive forms of the *E. coli* RNR (pdb: 6w4x and pdb: 3uus, respectively). The α-subunit is depicted in green and gray, and the β-subunit in pink and maroon. The active form is an α_2/β_2 complex and dATP binding triggers the formation of an inactive α_4/β_4 ring complex. The 90º rotation shows the canonical α/α dimer that exists in both complexes including dATP (spheres) bound to the cone domain.

(**C**) Surface representation of the dATP bound human RNR ring complex (pdb: 6aui). This complex is comprised of two different dimeric forms of the RNR α domain - a canonical dimer (right) and an ATP cone-mediated α-α dimer (below).

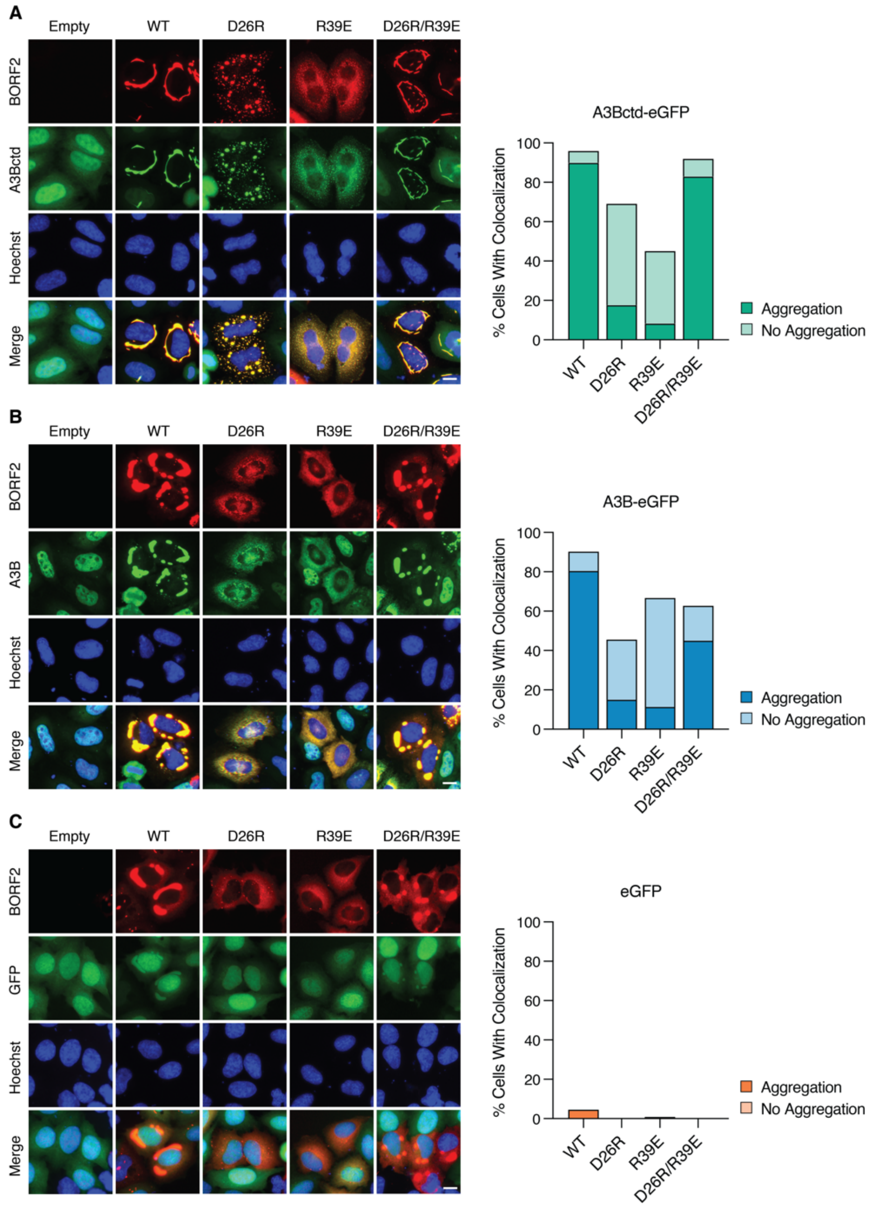

Fig. S9. Cellular localization profile of BORF2 with full-length A3B.
(**A**) Additional representative fluorescence microscopy images of A3Bctd-eGFP and the indicated mCherry-BORF2 constructs. Nuclei are stained with Hoechst (scale = 10µm). Right – histogram showing the percentage of cells with oblong BORF2-A3 aggregates (n > 50 for each condition).
(**B**) Representative fluorescence microscopy images of full-length A3B-eGFP and the indicated mCherry-BORF2 and constructs (scale = 10 µm). Nuclei are stained with Hoechst. Right – histogram showing the percentage of cells with aggregates and relocalized A3B-eGFP (n > 50 for each condition).
(**C**) Representative fluorescence microscopy images of eGFP and the indicated mCherry-BORF2 constructs (scale = 10 µm). Nuclei are stained with Hoechst. This control was included to show that some BORF2 aggregation occurs in the absence of A3B and that these structures are large enough to trap some eGFP. Right – histogram showing the percentage of cells with BORF2 aggregates (n > 50 for each condition).

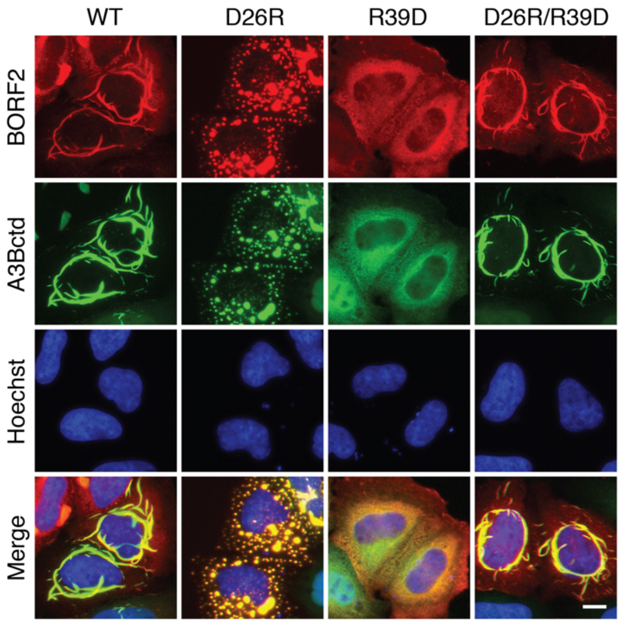

Fig. S10. Subcellular localization of noncanonical BORF2 dimerization interface mutants. Representative fluorescence microscopy images of A3Bctd-eGFP and the indicated mCherry-BORF2 constructs. The R39D mutants localize similar to the R39E mutants in the previous figure. Nuclei are stained with Hoechst (scale = 10µm).

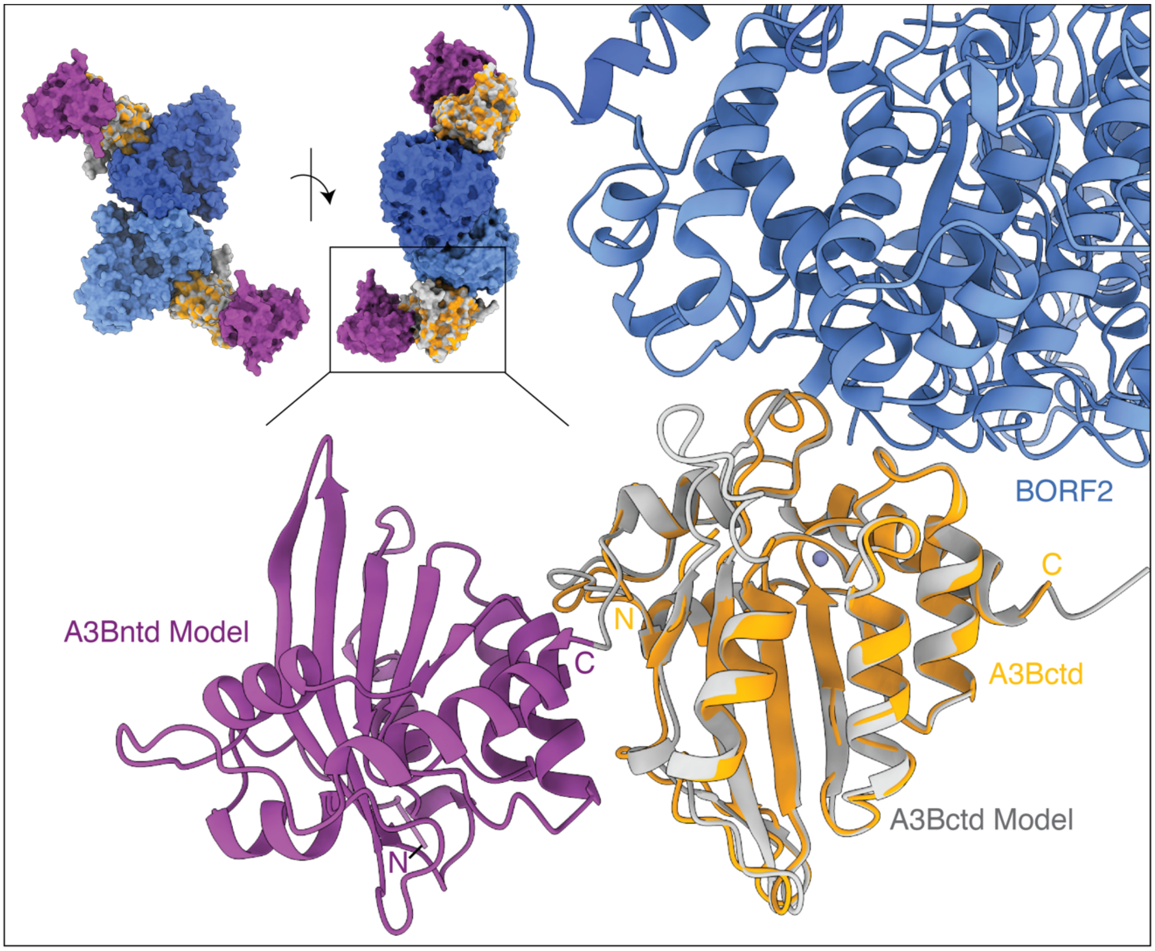

Fig. S11. Model of full-length A3B-BORF2 complex.
Surface representation and ribbon schematic of full-length A3B (purple and grey; downloaded from the AlphaFold Structure Database https://alphafold.ebi.ac.uk/) overlaid on the BORF2-A3Bctd cryo-EM structure (colored blue and orange as in other figures). The full-length A3B model is shaded such that the N-terminal domain (ntd) of A3B is purple and the C-terminal domain (ctd) is grey and placed over the actual A3Bctd structure in orange.

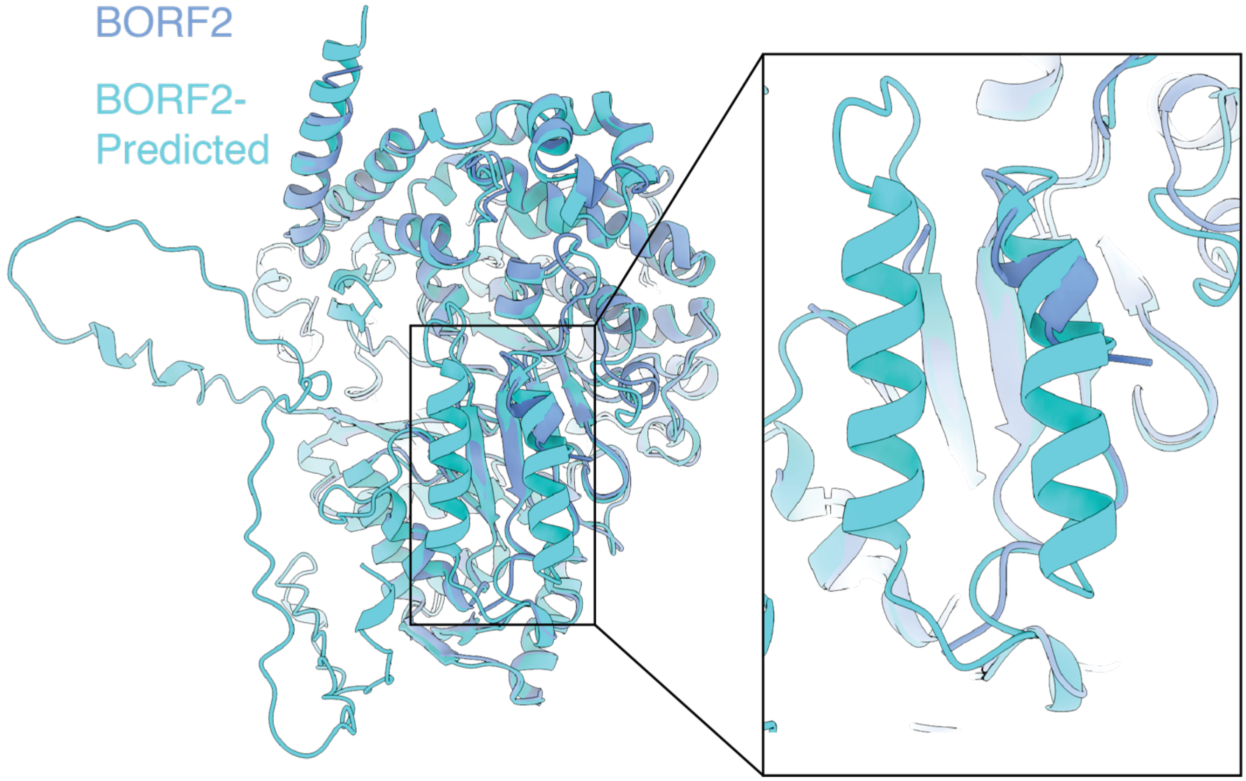

Fig. S12. The disordered canonical dimerization region in BORF2 is predicted to form α-helices.
Overlay of the BORF2 cryo-EM structure (darker blue) with the AlphaFold-predicted BORF2 structure (lighter blue). Right: close-up view of the predicted canonical dimerization region.

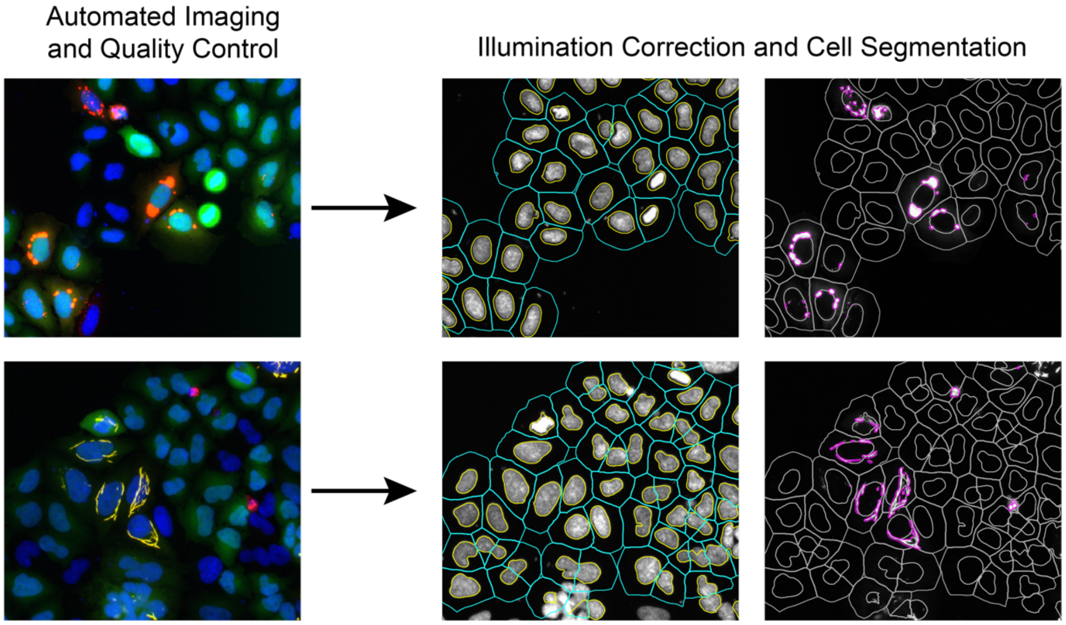

Fig. S13. Analysis pipeline for subcellular localization studies.
Representative images showing cell segmentation of nuclear and cytoplasmic compartments (middle; yellow and cyan tracing) and aggregate and oblong structures (right; pink tracing).

Table S1. Cryo-EM data collection and structure refinement data.

**Data collection and processing**			
Microscope		FEI Titan Krios	
Voltage (kV)		300	
Detector		Gatan K3	
Energy filter width (eV)		20	
Magnification (nominal X)		81,000	
Pixel size (Å)		0.844 (0.422 super-resolution)	
Defocus range (µm)		0.8-2.5	
Total Dose (e-/ $Å^2$)		60	
Number of frames		50	
Exposure time/movie (sec)		5.22	
Total Micrographs collected		7,488	
Micrographs used		6,759	
Automation Software		SerialEM	
Particles extracted (total)		7,675,126	
	**Overall**<br>**Global Resolution**	**BORF2-A3Bctd**<br>Focused-refinement around BORF2-A3B monomer	**Composite**<br>Generated from focused refined map using Phenix: combine focused maps
EMD #	EMD-24716	EMD-24715	EMD-24709
PDB #			7wr6
Particles	121,596	243,192 (Symmetry Expansion)	
Symmetry Imposed	C2	C1	
Map Sharpening B factor	-91.5	n/a DeepEMhancer	n/a DeepEMhancer
0.5 FSC (Unmasked/Masked) (Å)	6.03/3.33	4.31/3.01	
0.143 FSC (Unmasked/Masked) (Å)	3.84/2.82	3.6/2.55	
**Refinement and validation**			
Refinement package			Phenix
Refinement tool*			Real-space refine
Model composition			
Non-hydrogen atoms			13,130
Protein residues			1634
Ligands (Zn^{2+})			2
B factors ($Å^2$)			
Protein			73.1
Ligand			104.2
R.m.s deviations			
Bond lengths ($Å^2$)			0.003
Bond angles (º)			0.55
Ramachandran Plot			
Favored (%)			94.1
Allowed (%)			5.9
Disallowed (%)			0
Validation			
MolProbity score			1.89
Clashscore			9.15
Poor rotomers (%)			0
C-beta outliers (%)			0
CaBLAM outliers (%)			0.25
CC (mask)			0.87
EMRinger			4.11

Movie S1. 3D Variability analysis of BORF2-A3Bctd complex. Top view of the complex. This analysis was performed in cryoSPARC using the 3D variability feature.

Movie S2. 3D Variability analysis of BORF2-A3Bctd complex. Side view of the complex. This analysis was performed in cryoSPARC using the 3D variability feature.
